# R/G editing in GluA2R_flop_ modulates the functional difference between GluA1 flip and flop variants in GluA1/2R heteromeric channels

**DOI:** 10.1038/s41598-017-13233-2

**Published:** 2017-10-20

**Authors:** Wei Wen, Chi-Yen Lin, Li Niu

**Affiliations:** Department of Chemistry, and Center for Neuroscience Research, University at Albany, SUNY, Albany, New York, 12222 United States

## Abstract

In α-amino-3-hydroxy-5-methyl-4-isoxazole-propionate (AMPA) receptors, RNA editing and alternative splicing generate sequence variants, and those variants, as in GluA2-4 AMPA receptor subunits, generally show different properties. Yet, earlier studies have shown that the alternatively spliced, flip and flop variants of GluA1 AMPA receptor subunit exhibit no functional difference in homomeric channel form. Using a laser-pulse photolysis technique, combined with whole-cell recording, we measured the rate of channel opening, among other kinetic properties, for a series of AMPA channels with different arginine/glycine (R/G) editing and flip/flop status. We find that R/G editing in the GluA2 subunit modulates the channel properties in both homomeric (GluA2Q) and complex (GluA2Q/2R and GluA1/2R) channel forms. However, R/G editing is only effective in flop channels. Specifically, editing at the R/G site on the GluA2R flop isoform accelerates the rate of channel opening and desensitization for GluA1/2R channels more pronouncedly with the GluA1 being in the flop form than in the flip form; yet R/G editing has no effect on either channel-closing rate or EC_50_. Our results suggest R/G editing via GluA2R serve as a regulatory mechanism to modulate the function of GluA2R-containing, native receptors involved in fast excitatory synaptic transmission.

## Introduction

**Figure 1:**
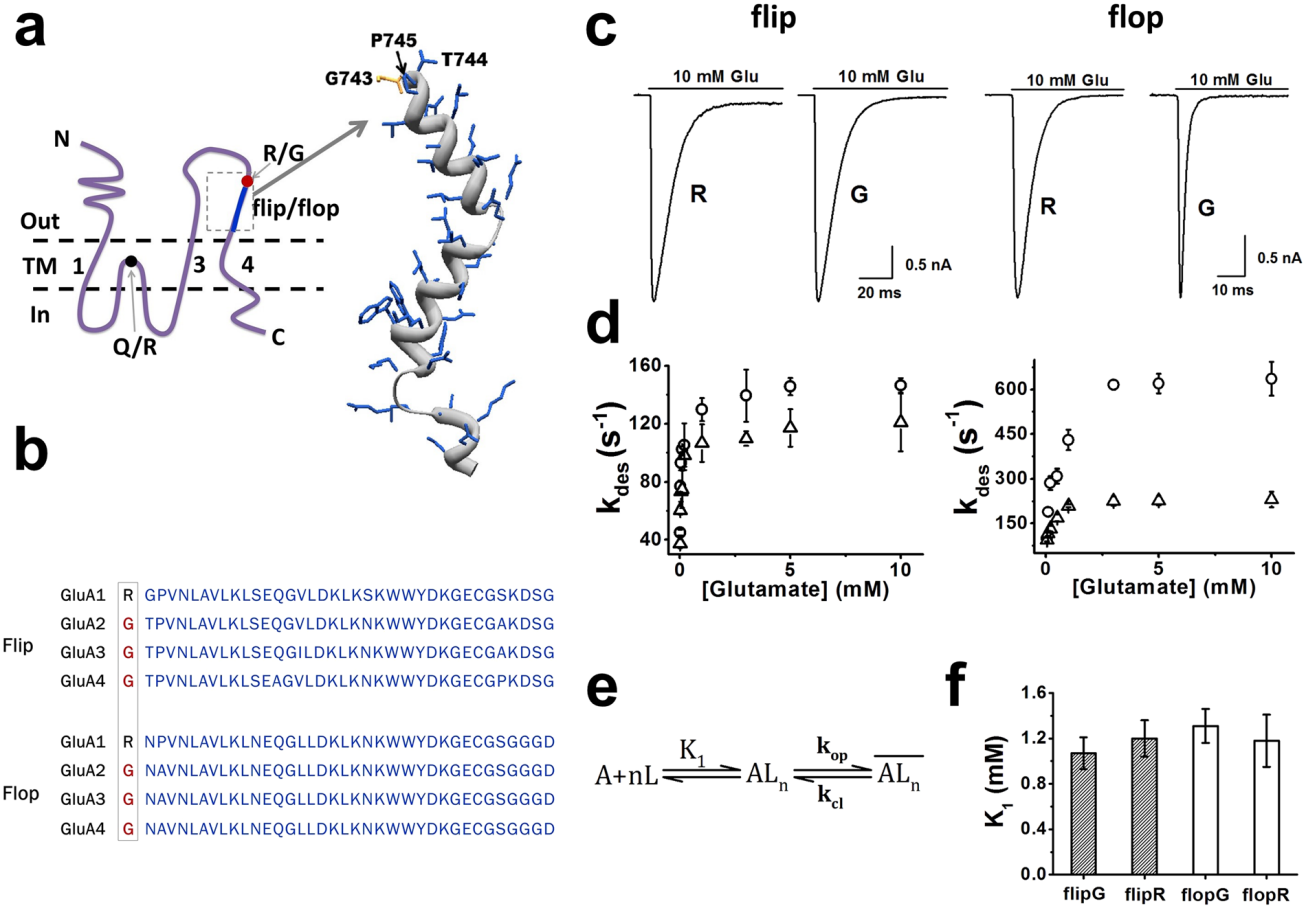
AMPA receptor sequences for R/G editing and flip/flop alternative splicing, GluA2 topology, and R/G editing effect on GluA2Q homomeric channel. (**a**) Schematic drawing of GluA2 subunit (left) and crystal structure of the R/G site and the flip/flop region (right, RCSB PDB: 3KG2). Each subunit consists of an extracellular N-terminal domain (N), a transmembrane domain (TM) that consists of three TM segments (TM1, 3, 4) and a re-entrant loop, and an intracellular C-terminal domain (C). Flip/flop alternative splicing cassette is labeled blue, and the R/G editing site is labeled red. (**b**) Sequence alignment of the R/G editing site and flip/flop sequence cassette of GluA1-4. R/G sites in GluA2-4 are boxed (GluA1 is not subject to R/G editing, and the equivalent position is an arginine). Complete sequences can be found in Genbank. (**c**) Representative whole-cell current responses of R/G edited and unedited GluA2Q_flip_ (left) and GluA2Q_flop_ (right) channels. (**d**) Dependence of desensitization rate on glutamate concentration for GluA2Q_flip_ (left) and GluA2Q_flop_ (right) channels edited (○) and unedited (Δ) at the R/G site. The maximum k_des_ for each of these channels is summarized in Table 1. (**e**) A general mechanism of channel opening for AMPA receptors. A represents a receptor at its resting state, L the ligand, n the number of ligand bound to the receptor, *AL*_*n*_ the closed-channel state with n ligands bound, $\overline{A{L}_{n}}$ the open-channel state. The value of n ranges from 1 to 4 due to that AMPA receptor is a tetramer and each subunit contains one ligand binding site, and k_op_ and k_cl_ stand for the channel-opening and channel-closing rate constant, respectively. It is also assumed that ligand binds with equal affinity or K_1_, the intrinsic equilibrium dissociation constant, at all steps (see all the equations derived from this mechanism and used for data analysis in Methods). (**f**) K_1_ values of GluA2Q_flip_ (shadowed) and GluA2Q_flop_ (hollow) channels edited (G) and unedited (R) at the R/G site. All the K_1_ values are determined from non-linear fitting using equation (3) (see Methods), and are summarized in Table 1 (Two-tailed Welch’s *t*-test; K_1_: flip(R) versus flip(G), *p* = 0.19, n = 3; K_1_: flop(R) versus flop(G), *p* = 0.13, n = 3).

AMPA receptors mediate the majority of excitatory synaptic transmission and are involved in the brain development and synaptic plasticity^1,2^. AMPA receptors have four subunits, i.e., GluA1-4, and each subunit is subject to RNA alternative splicing, generating the flip and flop variants. The flip and flop variants of GluA2-4 show distinct difference in channel opening rate^3^, desensitization rate^4–6^, and channel recovery rate^7,8^. In contrast, the GluA1 flip and flop variants (in homomeric channel forms) have identical kinetic properties^9–11^. It is seemingly paradoxical that GluA1 is “wired” with flip/flop sequence, like GluA2-4 (Fig. 1a,b); yet unlike GluA2-4, GluA1 defies one of the main intents of alternative splicing, i.e., expanding the functional diversity through alternative splicing^12^. Here, we set out a hypothesis by which the arginine/glycine (R/G) site on GluA2 regulates and diversifies the function of GluA1 flip and flop variants in the GluA1/2 heteromeric channel form. Our hypothesis is based on the following rationale.

AMPA receptors are expressed in almost all neurons involved in fast glutamatergic signaling in the central nervous system (CNS)^13–15^. Native AMPA receptors are assembled into tetramers possibly from GluA1-4 subunits^1^. GluA1/2 constitutes a major AMPA receptor population, although GluA1 can exist in homomeric channel form – in CA1/CA2 pyramidal neurons, GluA1 homomeric channel is thought to constitute ~8% of AMPA receptors^13^. GluA1/2 is especially rich in the rat hippocampus and cortex^13,16–18^. In extrasynaptic somatic AMPA receptors, ~95% are GluA1/2^17^. Furthermore, the GluA1 flop variant is expressed slightly higher than the flip throughout brain in both prenatal^19^ and postnatal periods^20,21^. The flip/flop ratio for GluA1 in the hippocampus is altered in some neurological disorders, such as seizure, in both animal models^22–24^ and patients^25^. The expression of GluA1 flip variant relative to the flop is found elevated in the nucleus accumbens and prefrontal cortex as a result of neonatal ventral hippocampal lesions in rats, an animal model for schizophrenia^26^, and in the striatum of the unilateral 6-hydroxydopamine (6-OHDA)-lesioned rats, an animal model for Parkinson’s disease^27^. These results show that the GluA1 flip/flop ratio is linked to both normal and abnormal activities of AMPA receptors involving most likely GluA1/2R. In fact, surface expression of GluA1/2 is increased in the nucleus accumbens of cocaine-sensitized rats^28^. Because the GluA1 flip and flop variants coexist, any functional difference between them in the GluA1/2 channel configuration could be significant.

RNA editing is another post-transcriptional RNA processing event that alters the nucleotide sequence of an RNA, relative to the sequence of the encoding DNA^29–31^. For example, adenosine to inosine (A-I) editing converts adenosine to inosine in double stranded RNAs by the action of adenosine deaminases that act on RNA (ADAR). A-to-I conversion is the most prevalent form of RNA editing in CNS^29,32^. In AMPA receptors, GluA2-4, but not GluA1, undergo A-I editing at two sites: the Q/R and R/G sites^33–35^. The Q/R editing site is exclusively found in the GluA2 subunit, and the Q/R editing efficiency is close to 100% in healthy adult human brain^33,36–38^. The unedited Q isoform of GluA2 undergoes forward trafficking and forms homomeric, high-conductance channels by itself. In contrast, the edited isoform or GluA2R is largely endoplasmic reticulum (ER)-retained and unassembled^39^. When expressed alone^40^, GluA2R channels exhibit femtoseimen conductance, and are Ca^2+^ impermeable^41^. Furthermore, when expressed in organotypic hippocampal slices, GluA2R alone shows no electrophysiological activity^16^. Yet GluA2R can be assembled with Q isoforms, and GluA2R-containing channels have either linear or outwardly rectifying current-voltage (I-V) relationships, high conductance but low Ca^2+^ permeability^7,42,43^. As a comparison, GluA2R-lacking receptors, i.e., homomeric and heteromeric AMPA receptors composed of only Q isoforms, all have high conductance, high Ca^2+^ permeability and inwardly rectifying I-V relationships^42,44–46^.

R/G editing occurs in GluA2-4, but not GluA1^33^. During development, the R/G site is generally edited differentially^33,38,47,48^. For example, the R/G site on GluA2 flip isoform is edited ~30% in the spinal cord but >70% in the cerebellum^48^. GluA2 and GluA3 are edited completely in the nucleus of the glossopharyngeal/vagal nerves, but only 60–80% in auditory structures^49^. R/G editing efficiency is linked to the mRNA level of ADAR2 and is further regulated by neural activities such as chronic depolarization/silencing^50,51^. In addition, R/G over-editing possibly underlies epileptic seizure^52^. Because R and G isoforms of GluA2 generally co-exist in the brain, any functional difference between the two isoforms could be biologically relevant.

Currently, R/G editing in GluA2Q is known to enhance the rate of channel desensitization and recovery from it, reduce the assembly of homomeric receptors, and slow receptor maturation in ER^33,53,54^. However, some major questions remain. Whether R/G editing affects the channel-opening properties of AMPA receptors and whether the effect of R/G editing correlates to alternative splicing that generates the flip/flop variants are unknown. If the R/G editing does regulate and expand the GluA1 function by separating the difference between the GluA1 flip and flop variants, it will be the R/G editing site on GluA2R that exerts that regulatory function. This is because normally, postnatal GluA2 exists virtually only in the Q/R-edited or R isoform. To test the hypothesis, we have characterized the kinetic properties, including the channel-opening kinetic properties, of a series of GluA2 and GluA1/2R R/G editing variants in the background of flip/flop splicing and Q/R editing variants. For measuring the rate of channel opening, we used a laser-pulse photolysis technique with a caged glutamate [e.g., *γ*-*O*-(*α*-carboxy-2-nitrobenzyl)glutamate]^55^, combined with whole-cell current recording. This technique enabled us to measure the rate of channel opening with a ~60 microsecond (µs) time resolution^55–57^.

## Results

### Experimental Design

To test the hypothesis in which R/G editing on GluA2 regulates and diversifies the function of GluA1 flip and flop variants in the GluA1/2R heteromeric channel complexes, we designed a study that included four homomeric (i.e., GluA2Q channels), eight GluA2Q/2R channels and eight GluA1/2R heteromeric channels. These channel forms contained different flip and flop combinations; in GluA2, there were two Q/R editing and two R/G editing isoforms as well. For studying the R/G editing of GluA2R, our design included three steps, due to the fact that expression of GluA2R alone has not been shown to produce any appreciable whole-cell current^58^. We started with GluA2Q channels with both R and G editing isoforms, together with the flip and flop variants. GluA2Q isoform is known to assemble into functional channels when expressed in a heterologous system such as HEK-293 cells. We wanted to first determine whether R/G editing affected the kinetic properties of the flip and flop isoforms of GluA2Q. The specific channel properties we characterized included channel-opening and channel-closing rate constants (k_op_ and k_cl_), along with desensitization rate constant (k_des_), EC_50_ and K_1_, the intrinsic equilibrium dissociation constant (see Methods). These constants define the basic gating property of these channels. To keep track of various combinations, we use the following nomenclature. For example, GluA2Q(G)_flip_ refers to the channel formed by GluA2 flip variant that is unedited at the Q/R site (i.e., Q) but edited at the R/G site (i.e., G). Next, we characterized the GluA2Q/2R channel with various R/G editing combinations, based on the following reasons. To date, whether R/G editing affects GluA2R is unclear. Knowing whether R/G editing affects GluA2R is essential in understanding the effect of the R/G editing of GluA2R on other GluA2R-containing channels, such as GluA1/2R. By using the same subunit and the same flip/flop variant (*since GluA2Q and 2R share the same flip/flop sequence module*), any potential change in the receptor properties, as compared with those of GluA2Q homomeric channels, can be attributed to the R/G editing status, rather than variation of the flip/flop sequences. In this sense, the study of GluA2Q flip and flop channels served as the control for the study of GluA2Q/2R channels. The outcome of these experiments was to determine whether R/G editing in GluA2R had any functional role in the GluA2Q/2R channels. Finally, we investigated the effect of R/G editing in the GluA2R subunit on the GluA1 flip and flop variants in the GluA1/2R channel form.

### R/G Editing Accelerates Desensitization of GluA2Q_flop_ Channel

To characterize whether the effect of R/G editing is flip/flop-dependent, we mutated the Gly at position 743 (R/G site) to Arg in both the flip and the flop wild-type GluA2Q isoforms, and expressed these isoforms individually in HEK-293 cells. We first measured the rate of channel desensitization as a function of glutamate concentration (i.e., from 50 μM to 20 mM) (Fig. 1c,d). The R/G edited channels, i.e., GluA2Q(G)_flip_ and GluA2Q(G)_flop_, desensitized with a first-order rate constant similar to those previously published^10,56,59^. As seen, R/G editing accelerated the rate of desensitization for both the flip and the flop GluA2Q channels. However, the maximum k_des_ of the R/G edited flop variant was 2.8-fold larger than that of the unedited flop isoform (Fig. 1d, right panel). In contrast, the R/G edited flip variant showed only a 1.2-fold higher k_des_ than the unedited flip channel (Fig. 1d, left panel). These data showed the effect of R/G editing on the increase of k_des_ was more significant in the flop background of GluA2Q (Fig. 1c,d; Table 1).

**Table 1:** Effects of R/G Editing on GluA2Q Channel Gating Properties.

GluA2Q(X)^a^		k_des_ (s^−1^)^b^	EC_50_ (mM)^c^	K_1_ (mM)^c^	k_op_ (×10^4^ s^−1^)^c^	k_cl_ (×10^3^ s^−1^)^c^
flip	R	121 ± 20	1.13 ± 0.05	1.20 ± 0.16	5.8 ± 0.5	1.5 ± 0.1
	G	146 ± 5^d^	1.04 ± 0.04	1.07 ± 0.14	5.5 ± 0.4	1.5 ± 0.1
flop	R	231 ± 26	1.27 ± 0.03	1.18 ± 0.23	5.2 ± 0.4	2.0 ± 0.1
	G	636 ± 57^d^	1.33 ± 0.07	1.31 ± 0.15	5.2 ± 0.5	3.4 ± 0.1^d^

^a^The X indicates the editing status of the R/G site.

^b^The k_des_ values are means (±SD) of those obtained from the saturated whole-cell responses evoked by 10 and 20 mM glutamate.

^c^K_1_, k_op_, k_cl_, and EC_50_ values (±SEM) were yielded through fitting (See Results).

^d^Significantly different from the corresponding unedited R variant. Comparisons of the parameters were made with two-tailed Welch’s *t-*test assuming population variances were not equal. Differences with *p* ≤ 0.05 were considered significant.

### R/G Editing Does Not Affect the Dose-Response Relationship of GluA2Q Homomeric Channels

The R/G editing site is located in helix J that lines the dimer interface of the ligand-binding domain, making it possible that the editing at this site could affect ligand binding^60^. Previous studies have also suggested that some of the R/G-edited channels show higher EC_50_ values^33^. Therefore, we determined the EC_50_ value for all four GluA2Q channels, i.e., GluA2Q(R)_flip_, GluA2Q(R)_flop_, GluA2Q(G)_flip_ and GluA2Q(G)_flop,_ from the fitting of the dose-response relationship (Table 1; a representative fit is shown in Supplementary Fig. S1a). Our results showed R/G editing did not affect EC_50_ of these GluA2Q receptors (Table 1). We also analyzed these dose-response relationships using a minimal, general model of channel opening (Fig. 1e; Methods) in order to obtain K_1_ (Supplementary Fig. S1a; Supplementary Table S1a). By this model, the K_1_ values for all four GluA2Q channels were also identical, within experimental error, suggesting that R/G editing did not affect K_1_ (Table 1). Similarly, both the K_1_ and EC_50_ values determined here are in good agreement with those we published previously on the edited isoform of GluA2Q_flip_ and GluA2Q_flop_ channels^10,56^. Based on these results, R/G editing did not affect the dose-response relationship of the GluA2Q receptors, regardless of whether a receptor was in the flip or flop isoform (Fig. 1f; Table 1). Furthermore, EC_50_ and K_1_ values are numerically similar, as seen in Table 1. Because K_1_ is defined as the intrinsic equilibrium dissociation constant, that K_1_ was unaffected by the R/G editing in either the flip or the flop background suggested that R/G editing did not affect the ligand binding affinity.

### R/G Editing Increases the Channel-closing Rate Constant of GluA2Q_flop_, but not GluA2Q_flip_, Channel

**Figure 2:**
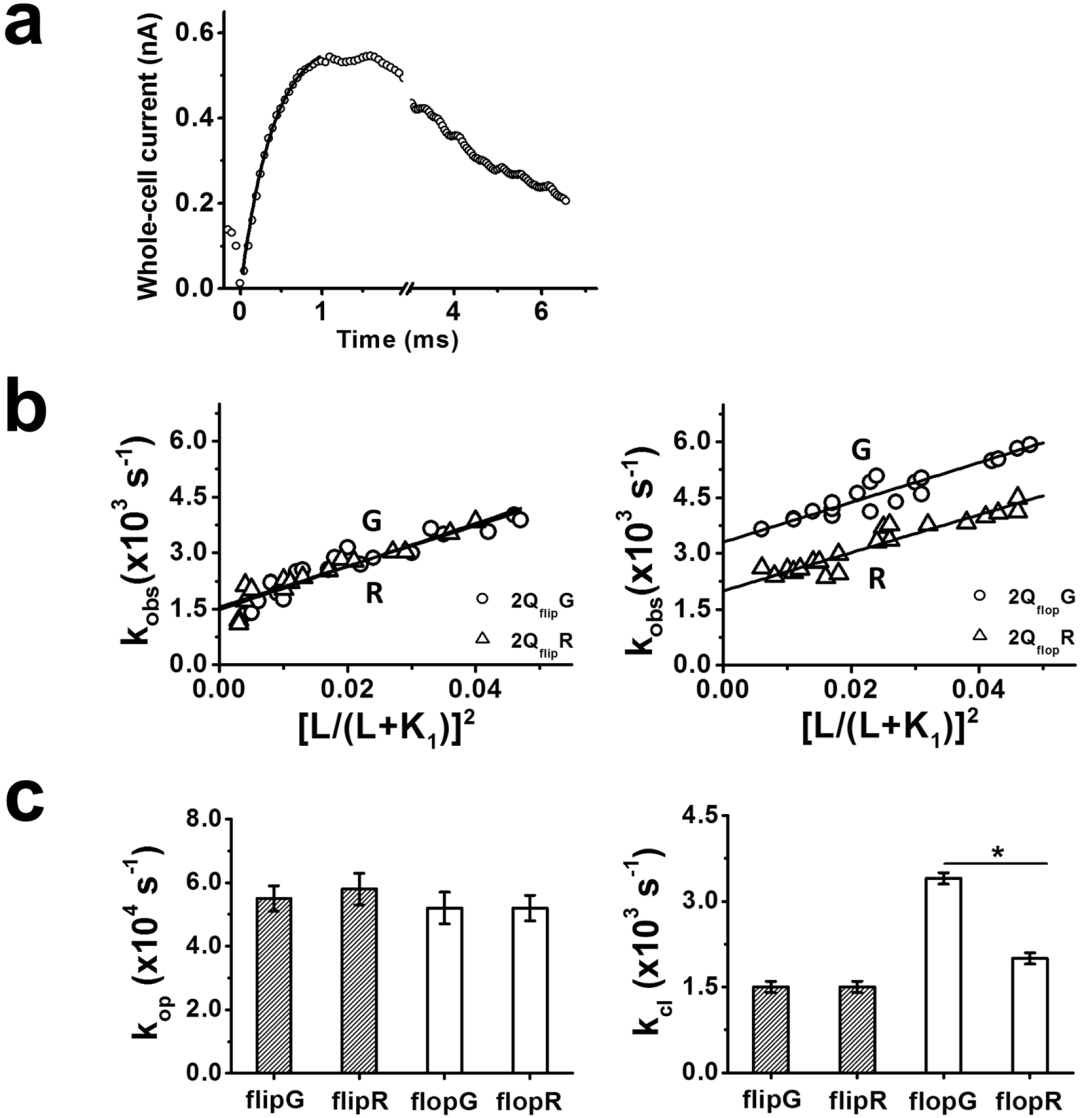
Channel opening kinetics of R/G edited and unedited GluA2Q homomeric channels obtained from laser-pulse photolysis measurement. (**a**) A representative whole-cell current trace from the opening of GluA2Q(R)_flip_ channels initiated by a laser-pulse photolysis of caged glutamate at time zero. For clarity of illustration, the number of data points was reduced in the rising phase of the current. The observed channel opening rate constant, k_obs_, was determined by fitting the rising phase to a single-exponential rate expression (solid line) using equation (1). (**b**) Linear fit of k_obs_ as a function of glutamate concentration (by equation (2)) for GluA2Q_flip_ (left) and GluA2Q_flop_ (right) channels edited (G, ○) and unedited (R, Δ) at the R/G site, respectively. Each data point represents a k_obs_ value obtained at a particular concentration of photolytically released glutamate. (**c**) The left and right panels show, respectively, the k_op_ and k_cl_ values of GluA2Q_flip_ (shadowed) and GluA2Q_flop_ (hollow) channels edited (G) and unedited (R) at the R/G site obtained from the linear fitting in Fig. 3b (Two-tailed Welch’s *t*-test; k_op_: flip(R) versus flip(G), *p* = 0.41, n = 3; k_op_: flop(R) versus flop(G), *p* = 0.37, n = 3; k_cl_: flip(R) versus flip(G), *p* = 0.44, n = 3; k_cl_: flop(R) versus flop(G), *p* ≤ 0.05, n = 3). All of the rate constants are summarized in Table 1. *p ≤ 0.05.

Using the laser-pulse photolysis technique combined with whole-cell recording, we investigated whether R/G editing affected the rate of channel opening of the GluA2Q channels. A representative whole-cell current response to glutamate, liberated by the laser-pulse photolysis, showed a first-order rate process for ~95% of the rising phase (i.e., the solid line in Fig. 2a). The observed channel-opening rate constant (k_obs_) was obtained from a first-order exponential fit of the current rise to equation (1) (Methods). From the best fit of k_obs_ as a function of the concentration of photolytically released glutamate, k_op_ and k_cl_ were estimated using equation (2) at n = 2; n is the number of ligands or glutamate molecules that bind to and open the channel (k_op_ and k_cl_ values are summarized in Table 1). Here, n = 2 was the best fit based on non-linear regressions using equation (2) (Supplementary Tables S2a–d, 3a–d, 4a) for both the flip and flop R/G channels, consistent with our previous finding that two glutamate molecules per channel are minimally required to open the channel and that higher receptor occupancy (n = 3 or 4) does not give rise to different k_op_ values^61^. Comparison of these rate constants showed that R/G editing did not affect k_op_ in either the flip or the flop variant (see slope in Fig. 2b; Fig. 2c left panel; Table 1; Supplementary Table S2a; Supplementary Figs S3a–h, 4a). Yet editing at the R/G site did increase k_cl_ by 1.7-fold for GluA2Q(G)_flop_ as compared with GluA2Q(R)_flop_ channel. In contrast, editing did not affect k_cl_ for the flip channels (Fig. 2c right panel; Table 1). Since k_cl_ = 1/τ, and τ is the lifetime, a larger k_cl_ or a faster channel closing rate suggested that editing at the R/G site turned the open channel of the flop isoform less stable. In addition, K_1_ was independently estimated using nonlinear regression of the channel-opening rate data. We found K_1_ of 1.1 mM on average (Supplementary Table S3a–h) was in good agreement with K_1_ of 1.2 mM estimated from the dose-response data (Supplementary Table S1a).

### R/G Editing Differentially Affects Desensitization Rate for GluA2Q/2R Channels

The percentage of R/G editing of AMPA receptors generally ranges from 45% to 60% in different subregions of mammalian brain^52,62,63^, suggesting the R/G unedited and edited isoforms at the subunit level (i.e., GluA2-4) co-exist. In an attempt to characterize the potential functional differences among various isoforms and investigate the functional role of GluA2R, we next studied the GluA2Q/2R channels. All the combinations in the GluA2Q/2R channels are named, following the same nomenclature, for R/G unedited, partially edited and completely edited GluA2Q/2R channels.

**Figure 3:**
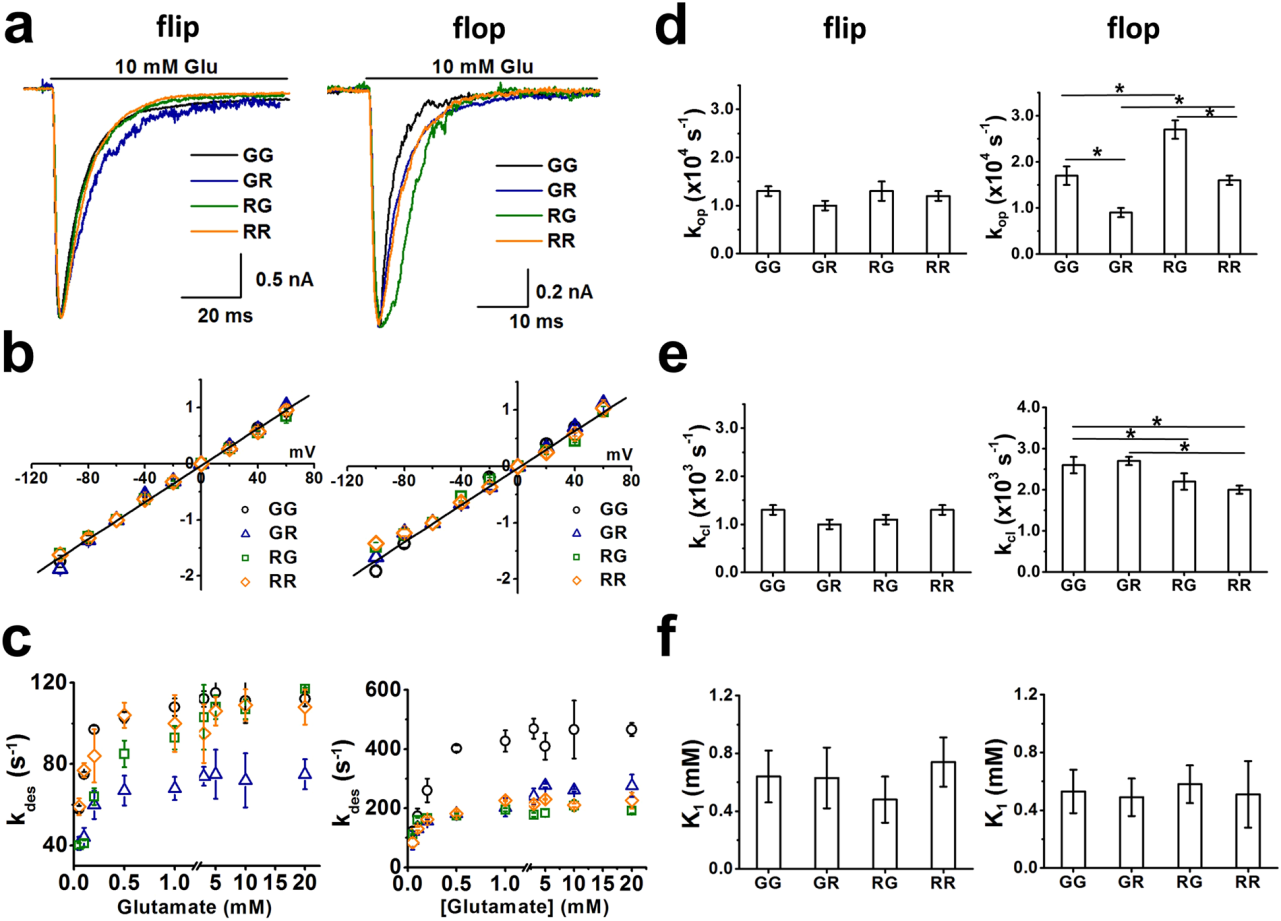
Kinetic properties of GluA2Q/2R complex channels with different R/G editing status. (**a**) Representative whole-cell current traces (10 mM glutamate) from GluA2Q_flip_/2R_flip_ (left) and GluA2Q_flop_2R_flop_ (right) channels with four different R/G editing combinations, labeled in different colors and letters. For example, GR refers to the channel that contains the R/G edited in the GluA2Q and R/G unedited in the GluA2R isoforms (these are the same labels in Table 2). (**b**) Current-voltage relationships for GluA2Q_flip_/2R_flip_ and GluA2Q_flop_/2R_flop_ with different R/G editing status. Peak current amplitudes were measured from HEK-293 cells voltage clamped from −100 mV to +60 mV), and were normalized to current amplitudes at −60 mV. **(c)** Dependence of desensitization rate (k_des_) on glutamate concentration for via GluA2Q_flip_/2R_flip_ (left) and GluA2Q_flop_/2R_flop_ (right) channels. **(d)** The k_op_ values of GluA2Q_flip_/2R_flip_ (left) and GluA2Q_flop_/2R_flop_ (right) channels, full pairwise comparisons were performed for flip and flop, respectively. (One-way ANOVA with Tukey’s correction; flip: *p* = 0.77, F_3,5_ = 3.96; flop(RR) versus flop(RG), *p* ≤ 0.05, n = 3; flop(RR) versus flop(GR), *p* ≤ 0.05, n = 3; flop(RR) versus flop(GG), *p* = 0.98, n = 3; flop(RG) versus flop(GR), *p* ≤ 0.05, n = 3; flop(GG) versus flop(RG), *p* ≤ 0.05, n = 3; flop(GG) versus flop(GR), *p* ≤ 0.05, n = 3). **(e)** The k_cl_ values of GluA2Q_flip_/2R_flip_ (left) and GluA2Q_flop_/2R_flop_ (right) channels, full pairwise comparisons were performed for flip and flop, respectively. (One-way ANOVA with Tukey’s correction; flip: *p* = 0.73, F_3,5_ = 4.33; flop(RR) versus flop(RG), *p* = 0.60, n = 3; flop(RR) versus flop(GR), *p* ≤ 0.05, n = 3; flop(GG) versus flop(RG), *p* ≤ 0.05, n = 3; flop(GG) versus flop(GR), *p* = 0.35, n = 3;flop(GG) versus flop(RR), *p* ≤ 0.05, n = 3; flop(GR) versus flop(RG), *p* ≤ 0.05, n = 3). **(f)** K_1_ values of GluA2Q_flip_/2R_flip_ (left) and GluA2Q_flop_/2R_flop_ (right) channels. All the K_1_ values are determined from non-linear fitting of the dose-response data to equation, fittings are shown in Supplementary Fig. S1b,c. (3) (One-way ANOVA with Tukey’s correction; flip, *p* = 0.60, F_3,5_ = 4.47; flop, *p* = 1.01, F_3,5_ = 4.88). The detailed linear fittings in both (d) and (e) are provided in Supplementary Figs. S2 and S3. All of the constants are summarized in Table 2. *p ≤ 0.05.

A set of representative whole-cell current traces for the flip and flop Glu2Q/2R channels is shown in Fig. 3a. The formation of GluA2Q/2R channels in HEK-293 cells was confirmed by a linearized I-V curve (Fig. 3b)^42,44,64,65^. It should be noted that transfection of the GluA2R plasmid alone even at very high amount (30 µg per 35 mm Petri dish) did not produce HEK-293 cells that responded to glutamate^58^. We also individually expressed the flip and flop versions of the GluA2R(R) and GluA2R(G) in HEK-293 cells, but observed no appreciable whole-cell current response to glutamate at saturating concentrations (we tested ~30 cells for each type). Our observation agreed with the earlier finding that GluA2R cannot form a high conductance channel alone^40,58^.

Our measurement of channel desensitization showed several distinct features. (i) Regardless of the R/G editing status, flop channels desensitized faster than the flip counterparts (right panel vs. left panel in Fig. 3c). (ii) In flop channels, partially edited (GR and RG) and unedited (RR) channels desensitized with similar rates (Fig. 3c right panel; Table 2), whereas the completely R/G edited flop channel (GG, black symbol, right panel of Fig. 3c) desensitized 1.9-fold faster than any other channels (GR, RG, RR). (iii) In the flip channel category, the GR channel, i.e., GluA2Q(G)_flip_/GluA2R(R)_flip_, showed a ~1.5-fold slower channel desensitization rate (royal blue symbol in Fig. 3c, left panel; Table 2), as compared with GG, RR and RG channels. We do not yet know the reason for this difference at the present. Yet, when all four subunits are in the R/G edited isoform, GluA2Q/2R (Fig. 3c right panel), just like GluA2Q homomeric channel (Fig. 1d right panel), exhibits the fastest desensitization rate. In other words, the flop status at the individual subunit level is important for the effect of R/G editing.

**Table 2:** Effects of R/G Editing on GluA2Q/2R Channel Gating Properties.

GluA2Q(X)/2R(Y)^a^		k_des_ (s^−1^)^b^	EC_50_ (mM)^c^	K_1_ (mM)^c^	k_op_ (×10^4^ s^−1^)^c^	k_cl_ (×10^3^ s^−1^)^c^
flip	RR	110 ± 8^e^	0.82 ± 0.07	0.74 ± 0.17	1.2 ± 0.1	1.3 ± 0.1
	GR	75 ± 12	0.84 ± 0.15	0.63 ± 0.21	1.0 ± 0.1	1.0 ± 0.1
	RG	117 ± 1^e^	0.63 ± 0.05	0.48 ± 0.16	1.3 ± 0.2	1.1 ± 0.1
	GG	115 ± 6^e^	0.62 ± 0.06	0.64 ± 0.18	1.3 ± 0.1	1.3 ± 0.1
flop	RR	230 ± 2	0.50 ± 0.03	0.51 ± 0.23	1.6 ± 0.1	2.0 ± 0.1
	GR	277 ± 6	0.56 ± 0.05	0.49 ± 0.13	0.9 ± 0.1^d,f^	2.7 ± 0.1^d,f^
	RG	210 ± 18	0.63 ± 0.06	0.58 ± 0.13	2.7 ± 0.2^d,e^	2.2 ± 0.2
	GG	466 ± 22^d,e,f^	0.52 ± 0.07	0.53 ± 0.15	1.7 ± 0.2^e,f^	2.6 ± 0.2^d,f^

^a^X and Y indicate the R/G editing status in GluA2Q and GluA2R, respectively.

^b^The k_des_ values are means (±SD) of those obtained from the saturated whole-cell responses evoked by 10 and 20 mM glutamate.

^c^K_1_, k_op_, k_cl_, and EC_50_ values (±SEM) were yielded through fitting (See Results).

^d^Significantly different from the corresponding non-edited RR variant.

^e^Significantly different from the corresponding partially edited GR variant.

^f^Significantly different from the corresponding partially edited RG variant.

Comparisons of the parameters were made with one-way ANOVA with *post hoc* Tukey’s correction. Differences with *p* ≤ 0.05 were considered significant.

### R/G Editing Differentially Affects Channel-Opening Kinetics of GluA2Q/2R Channels

Using the laser-pulse photolysis technique, we measured the rate of channel opening of GluA2Q/2R with different R/G editing combinations. Similar to the way described for GluA2Q channels, we analyzed k_obs_ as a function of glutamate concentration and determined both the k_op_ and k_cl_ values for each of these channels (these constants are summarized in Table 2; see also Supplementary Tables S2b, 3i–l, 4b; fittings are shown in Supplementary Figs. S2 and S3). The data have allowed us to draw several conclusions. (i) R/G editing affected the flop channels (Table 2; Supplementary Table S4b), similar to its effect on the GluA2Q homomeric channels, but not the flip channels (left panel of Fig. 3d,e; Table 2; Supplementary Table S4b). (ii) When the unedited (RR) receptor is compared with the completely edited (GG) one (Fig. 3d right panel; Table 2), k_op_ is unaffected but k_cl_ is slightly higher for the GG channel (i.e., 2.6 × 10^3^ s^−1^ vs. 2.0 × 10^3^ s^−1^). This result is similar to that of the GluA2Q homomeric channel (Table 1). Interestingly, the average k_cl_ of the flop group is ~2-fold larger than that of the flip group (Fig. 3e, right vs. left panel; Table 2), while k_op_ values between these two groups are generally similar. Therefore, the difference in k_cl_ should be attributed to the flip/flop sequence module, rather than R/G editing. In fact, the same conclusion can be drawn as well, if, for example, GG flip channels are compared with the GG flop channels (Table 2). (iii) We also compared the K_1_ value of all GluA2Q/2R channels but found that all channels, regardless of their R/G editing status, exhibited similar K_1_ values, indicating similar ligand binding affinity (Fig. 3f; Table 2, Supplementary Fig. S1b,c). In addition, from either rate (Supplementary Table S3i–l) or amplitude (Supplementary Table S1b) measurements, our data showed that the minimal number of ligands that bound to and opened a GluA2Q/2R channel was two, regardless of the R/G editing status (Supplementary Tables S1b, 3i–l, 4b). This is also consistent with conclusion from our current and previous characterizations of the GluA2Q homomeric channels^56^.

The results from the study of the GluA2Q/2R complex channel clearly showed that the R/G editing on GluA2R in the flop background modulates the channel activity. When GluA2Q is in the R/G unedited state, editing at the R/G site on GluA2R changes k_op_ from 1.6 × 10^4^ s^−1^ to 2.7 × 10^4^ s^−1^ without affecting k_cl_ and k_des_ (Table 2). However, when GluA2Q is in the R/G edited state, upon R/G editing in GluA2R, k_op_ increases from 0.9 × 10^4^ s^−1^ to 1.7 × 10^4^ s^−1^, while k_cl_ is slightly elevated and k_des_ goes up from 277 s^−1^ to 466 s^−1^ (Table 2). More importantly, when the flip and flop groups are compared together, k_op_, k_cl_ and k_des_ of the flop variants are all larger. Based on these results, we predicted that the R/G editing in the GluA2R flop background would lead to a functional difference between the flip and flop variants of the GluA1 subunit in the GluA1/2R heteromeric channel configuration.

### The Flip/flop Variants of GluA1 Show Their Functional Divergence When Combined with GluA2R_flop_

**Figure 4:**
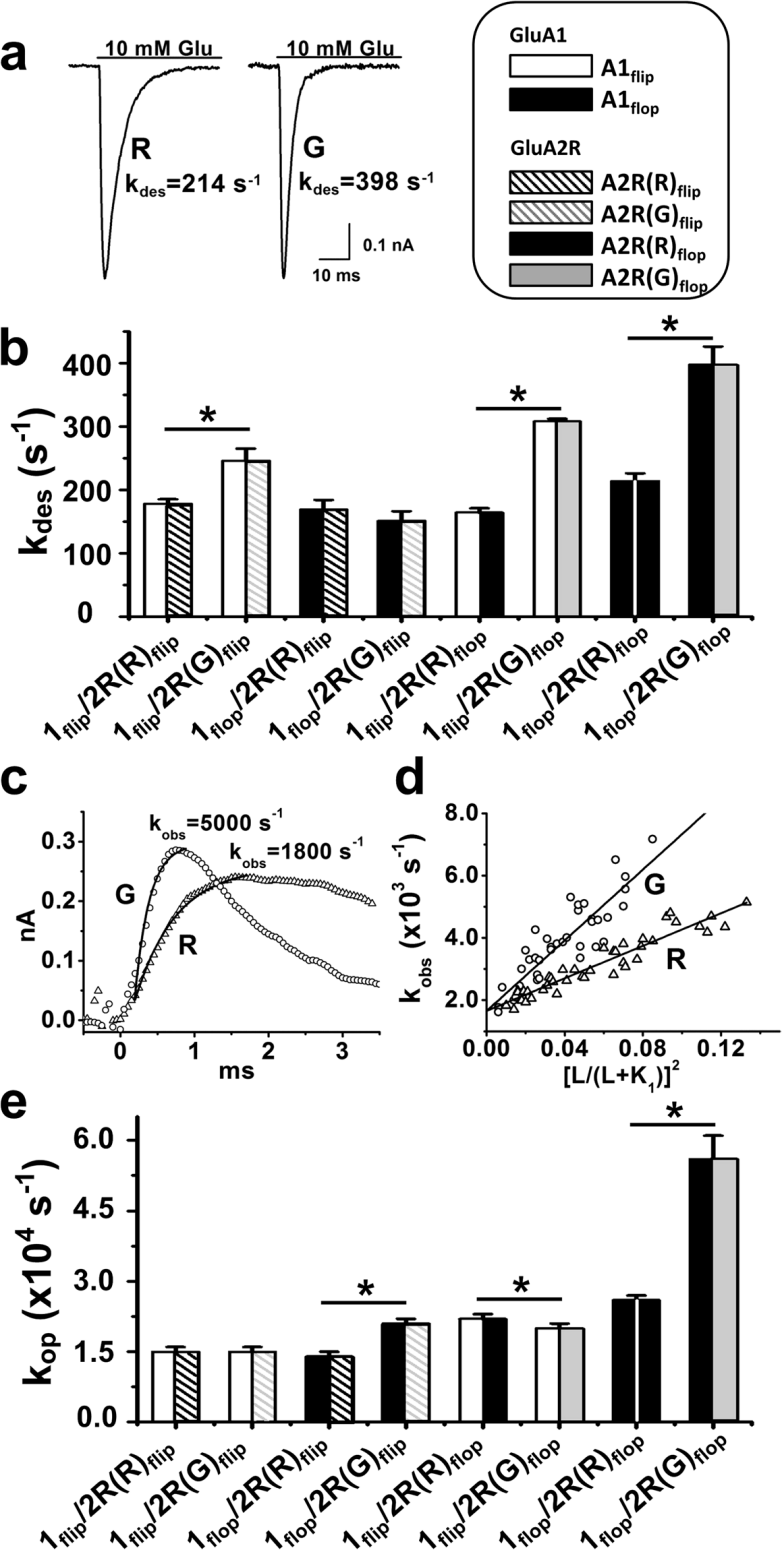
Effect of the R/G editing in GluA2R on the kinetic properties of GluA1/2R heteromeric channels. (**a**) Representative whole-cell current responses of GluA1_flop_/2R(R)_flop_ (left) and GluA1_flop_/2R(G)_flop_ (right) channels to 10 mM glutamate. **(b)** Maximum k_des_ values (at saturated glutamate concentrations) of GluA1/2R with different flip/flop and R/G editing (in GluA2R) status. From left to right, GluA2R in the flip form is shown in columns 1–4 and the flop form is in columns 5–8. All the columns are also shown in specific patterns, and the bar codes are shown in the box on top of panel (b). One-way ANOVA with Tukey’s correction was done for columns 1–4 or group 1(flip group) and columns 5–8 or group 2 (flop group), respectively (n = 3). Each group was subjected to full pairwise comparisons; yet, for clarity, only those pairs that were statistically significant between R and G are labeled in the figure (Group 1: columns 1 and 2, *p* ≤ 0.05; columns 1 and 3, *p* = 0.75; columns 1 and 4, *p* = 0.54; columns 2 and 3, *p* ≤ 0.05; columns 2 and 4, *p* ≤ 0.05; columns 3 and 4, *p* = 0.36. Group 2: columns 5 and 6, *p* ≤ 0.05; columns 5 and 7, *p* ≤ 0.05; columns 5 and 8, *p* ≤ 0.05; columns 6 and 7, *p* ≤ 0.05; columns 6 and 8, *p* ≤ 0.05; columns 7 and 8, *p* ≤ 0.05). **(c**) A pair of representative whole-cell current traces from the opening of GluA1_flop_/2R(R)_flop_ and GluA1_flop_/2R(G)_flop_ channels initiated by a laser-pulse photolysis of caged glutamate at time zero. The observed channel opening rate constant, k_obs_, was determined by fitting the rising phase to a single-exponential rate expression (equation (1)), shown as the solid line. **(d**) Linear fit of k_obs_ as a function of glutamate concentration (by equation (2)) of GluA1_flop_/2R(R)_flop_ (R, Δ) and GluA1_flop_/2R(G)_flop_ channels (G, ○), respectively. The k_op_ and k_cl_ values are shown in Tables 3 and 4. (**e**) The k_op_ values of various GluA1/2R channels with different R/G editing and flip/flop status. From left to right, GluA2R in the flip form is shown in columns 1–4 while the flop form is in columns 5–8. One-way ANOVA with Tukey’s correction was done for columns 1–4 or group 1(flip group) and columns 5–8 or group 2 (flop group), respectively (n = 3). Each group was subjected to full pairwise comparisons; yet, for clarity, only those pairs that were statistically significant between R and G are labeled in the figure (Group 1: columns 1 and 2, *p* = 0.88; columns 1 and 3, *p* = 0.85; columns 1 and 4, *p* ≤ 0.05; columns 2 and 3, *p* = 0.78; columns 2 and 4, *p* ≤ 0.05; columns 3 and 4, *p* ≤ 0.05. Group 2: columns 5 and 6, *p* = 0.11; columns 5 and 7, *p* ≤ 0.05; columns 5 and 8, *p* ≤ 0.05; columns 6 and 7, *p* ≤ 0.05; columns 6 and 8, *p* ≤ 0.05; columns 7 and 8, *p* ≤ 0.05). The detailed linear fittings in (e) are provided in Supplementary Fig. S6. All the constants are summarized in Tables 3 and 4. *p ≤ 0.05.

**Figure 5:**
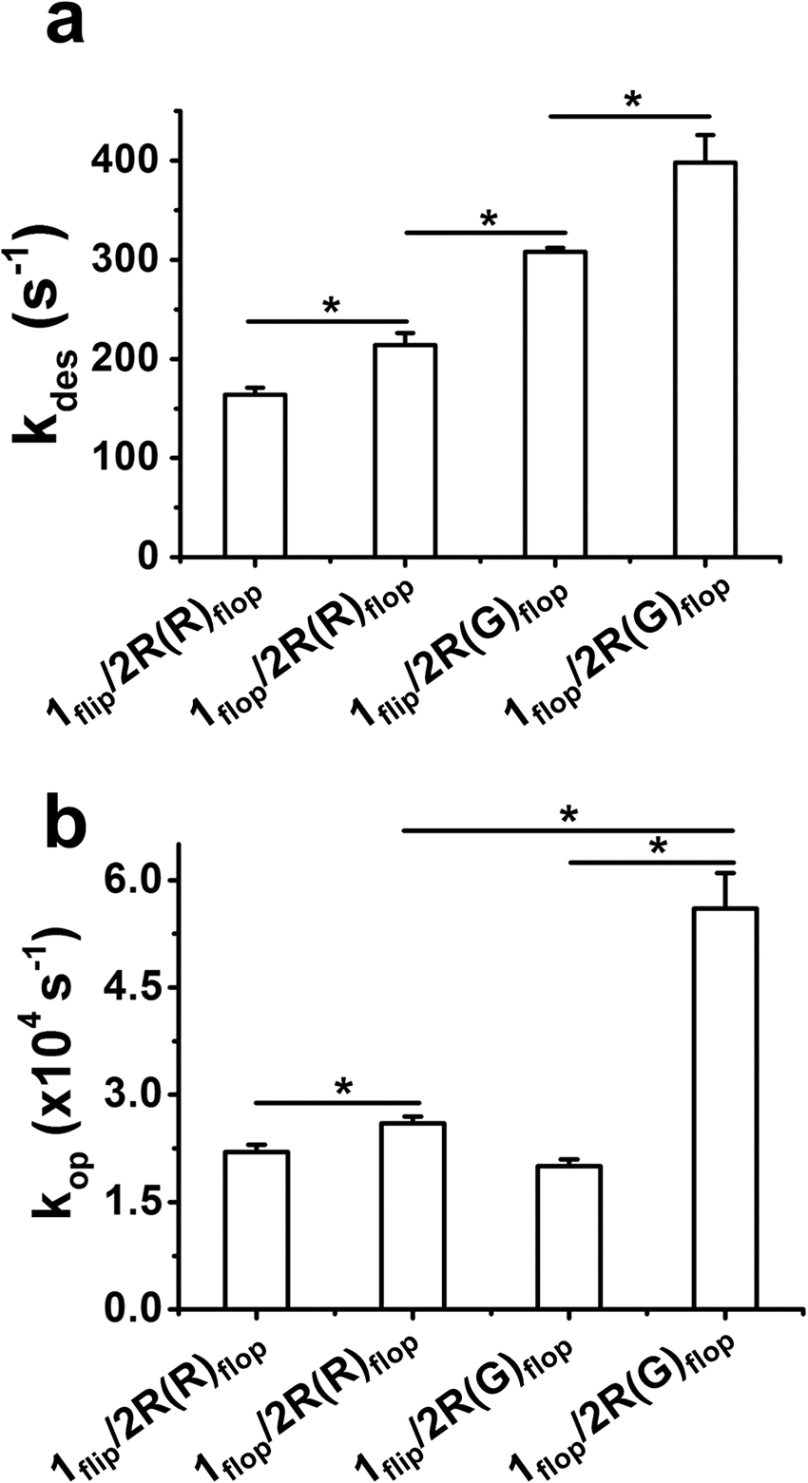
Effect of R/G editing on the GluA1/2R_flop_ channels with varying flip/flop contents in GluA1. Here, we compare k_des_ (**a**) and k_op_ (**b**) of GluA1/2R_flop_ receptors with the flip and flop GluA1 subunits; GluA2R is in the flop form with R/G unedited and edited isoforms (Two-tailed Welch’s *t*-test; k_des_: columns 1 and 2, *p* ≤ 0.05, n = 3; columns 2 and 3, *p* ≤ 0.05, n = 3; columns 3 and 4, *p* ≤ 0.05, n = 3; k_op_: columns 1 and 2, *p* ≤ 0.05, n = 3; columns 2 and 4, *p* ≤ 0.05, n = 3; columns 3 and 4, *p* ≤ 0.05, n = 3). *p ≤ 0.05.

Similar to the panel of the experiments we did for GluA2Q/2R, we characterized a series of GluA1/2R channels that combined various flip and flop variants of GluA1 and GluA2R, along with the R/G editing isoforms in GluA2R (the linear I-V curves for different GluA1/2R channels are shown in Supplementary Fig. S4 to confirm the formation of GluA1/2R channels). Figure 4a displays a pair of whole-cell current responses of GluA1_flop_/2R(R)_flop_ and GluA1_flop_/2R(G)_flop_ channels to 10 mM glutamate. As seen (Fig. 4a), the R/G edited channel in the flop background desensitized faster than the unedited channel; the maximum k_des_ value for various GluA1/2R channels are shown in Fig. 4b (see also Tables 3 and 4). For the comparison of all k_des_ values, eight GluA1/2R channels were divided into two groups based on the splicing status of the GluA2R subunit. In group 1 (columns 1–4 in Fig. 4b), all GluA2R were in the flip form; in group 2 (columns 5–8), all GluA2R were in the flop status. One-way ANOVA was performed for each group respectively, followed by full pairwise comparison using Tukey’s correction. Since we only attempted to understand, from this figure, whether R/G editing affected channel desensitization in both the flip and the flop background, for clarity, we only labeled those column that are statistically significant between R and G, e.g. columns 5 and 6, were labeled in the corresponding graph, although all the statistical values are provided in the Fig. 4b legend. Based on these data and statistical analysis, we concluded (i) when GluA2R is in the flip background, R/G editing has virtually no effect on k_des_ regardless of whether GluA1 is flip or flop status (Table 3; Fig. 4b, columns 1–4); (ii) R/G editing on GluA2R does affect k_des_ when GluA2R is in the flop background (Table 4; Fig. 4b, columns 5–8) (it should be noted that the detailed data analysis for channels with variable splicing status of GluA1 with GluA2R(R)_flop_ and GluA2R(G)_flop_ is presented in Fig. 5).

**Table 3:** Effects of R/G Editing in GluA2R_flip_ subunit on GluA1/2R Channel Gating Properties.

GluA1/2RX^a^		k_des_ (s^−1^)^b^	EC_50_ (mM)^c^	K_1_ (mM)^c^	k_op_ (×10^4^ s^−1^)^c^	k_cl_ (×10^3^ s^−1^)^c^	P_op_^e^
A1	2R(X)						
flip	(R)flip	178 ± 7	0.54 ± 0.15	0.57 ± 0.15	1.5 ± 0.1	1.3 ± 0.1	0.92
flip	(G)flip	246 ± 19^d^	0.62 ± 0.06	0.60 ± 0.17	1.5 ± 0.1	1.3 ± 0.1	0.92
flop	(R)flip	169 ± 15	0.63 ± 0.04	0.55 ± 0.46	1.4 ± 0.1	1.5 ± 0.1	0.90
flop	(G)flip	151 ± 15	0.83 ± 0.05	0.70 ± 0.49	2.1 ± 0.1^d^	1.6 ± 0.1	0.93

**Table 4:** Effects of R/G Editing in GluA2R_flop_ subunit on GluA1/2R Channel Gating Properties.

GluA1/2RX^a^		k_des_ (s^−1^)^b^	EC_50_ (mM)^c^	K_1_ (mM)^c^	k_op_ (×10^4^ s^−1^)^c^	k_cl_ (×10^3^ s^-1^)^c^	P_op_^e^
A1	2R(X)						
flip	(R)flop	164 ± 7	0.81 ± 0.11	0.71 ± 0.27	2.2 ± 0.1	1.9 ± 0.1	0.92
flip	(G)flop	308 ± 4^d^	0.63 ± 0.05	0.72 ± 0.35	2.0 ± 0.1	2.0 ± 0.1	0.91
flop	(R)flop	214 ± 12	0.56 ± 0.05	0.50 ± 0.17	2.6 ± 0.1	1.6 ± 0.1	0.94
flop	(G)flop	398 ± 28^d^	0.62 ± 0.07	0.70 ± 0.17	5.6 ± 0.5^d^	1.7 ± 0.2	0.97

X indicates the R/G editing status in GluA2R.

The k_des_ values are means (±SD) of those obtained from the saturated whole-cell responses evoked by 10 and 20 mM glutamate.

K_1_, k_op_, k_cl_, and EC_50_ values (±SEM) were yielded through fitting (See Results). P_op_ values were calculated by the equation P_op_ = k_op_/(k_op_ + k_cl_).

Significantly different from the corresponding non-edited R variant.

Comparisons of the parameters were made with one-way ANOVA with *post hoc* Tukey’s correction and Welch’s *t*-tests. Differences with *p* ≤ 0.05 were considered significant.

The analysis of the dose-response data allowed us to estimate both the EC_50_ and the K_1_ value for each of the GluA1/2R channels. Specifically, fitting of the dose-response data to the Hill equation (see Supplementary Fig. S5) yielded EC_50_ values, whereas fitting of the dose-response data to equation (3) (Methods) (see Supplementary Table S1c) gave us K_1_ and n, where n = 2 was the best fit. All the EC_50_ and K_1_ values are summarized in Tables 3 and 4. Our data and the data analysis have shown that the R/G editing in GluA2R does not affect either EC_50_ or K_1_ in all the GluA1/2R channel forms we tested (Tables 3 and 4). That the R/G editing had no effect on the dose-response relationship in GluA1/2R is further consistent with our observation in the study of both GluA2Q and GluA2Q/2R channels.

Using the laser-pulse photolysis technique, we characterized the effect of R/G editing in GluA2R on the channel-opening rate constant of all the GluA1/2R channels. Shown in Fig. 4c is a representative whole-cell current response to photolytically released glutamate from an HEK-293 cell expressing GluA1_flop_/2R(R)_flop_. Using equation (2), we analyzed k_obs_ vs. glutamate concentration by both linear and nonlinear regression (Supplementary Tables S3m–p, 4c; the methods and rationale of these analyses are similar to those used in the GluA2Q data, and are provided in the footnote in each of the Supplementary Tables). For example, we found the best fitted *n* value (the number of ligands that bind to the receptor to open the channel) was 2, and the K_1_ values the regression analysis returned (Supplementary Table S3m–p) were consistent with those we obtained from the dose-response data (Tables 3 and 4). A pair of representative, linear plots of the fitted values for both k_op_ and k_cl_ for the channels containing the R/G unedited and edited GluA2R is shown in Fig. 4d. For comparison, k_op_ values for all the GluA1/2R channel forms with different flip and flop as well as R/G editing isoforms are shown in Fig. 4e. Same data-grouping strategy as in Fig. 4b was used here. All of the k_op_ and k_cl_ data are summarized in Tables 3 and 4 (fittings are shown in Supplementary Fig. S6).

If all the data involving the flip GluA2R are grouped (Table 3; Fig. 4b,e) and compared, we can conclude that by and large, R/G editing in the GluA2R flip background had no or minimal effect on the kinetic constants of channel opening for GluA1/2R channels. In contrast, R/G editing clearly affected the properties of GluA1/2R_flop_ channels (Table 4; Fig. 4b,e). In Fig. 5a,b, we compared various channel configurations but with the flop isoform of GluA2R or precisely the R/G edited and unedited isoforms of GluA2R_flop_. Specifically, full pairwise comparisons show that as the number of flop subunit in the tetrameric complex becomes higher, the channel opens faster and desensitizes faster as well (Fig. 5a,b); and the channel open probability (P_op_) becomes larger (Table 4; Fig. 5b). In fact, k_des_, k_op_ and P_op_ are the largest when all four subunits are all flop and the GluA2R is R/G edited, namely, GluA1_flop_/GluA2R(G)_flop_ (Figs 4e and 5b). However, k_cl_, K_1_ and EC_50_ values remain the same for all of these heteromeric channel types (Tables 3 and 4). In a more detailed comparison, when GluA1 is in the flip form (GluA1_flip_/2R_flop_), the channel showed a 2-fold increase in k_des_ upon R/G editing in the GluA2R subunit (columns 1 and 3 in Fig. 5a; Table 4). Yet neither k_cl_ nor k_op_ was affected (columns 1 and 3 in Fig. 5b). When GluA1 is in the flop form (GluA1_flop_/2R_flop_), R/G editing led to ~2-fold increase in both k_des_ and k_op_, while k_cl_ still remains unaffected (Table 4; columns 2 and 4 in Fig. 5a,b). Taken together, these data and their comparisons have demonstrated that R/G editing in the GluA2R subunit plays a functional role in modulating the GluA1/2R channel activities; but minimally, the GluA2R subunit must be in the flop status. A more significant effect is seen when the GluA1 subunit is also in the flop variant in these GluA1/2R heteromeric channels.

## Discussion

R/G editing in AMPA receptors was first reported in 1994^33^, but the understanding of its role in regulating the gating properties, presumably by expanding the functional repertoire of the AMPA receptors, has been largely limited to a slow time scale. Here we have designed a comprehensive study that included 20 homomeric and heteromeric AMPA receptors that combine R/G editing isoforms with Q/R editing and flip/flop RNA splicing variants. Using a laser-pulse photolysis technique that provided ~60 µs time resolution^3,10,56,66,67^, we have systematically investigated the effect of R/G editing on the channel-opening rate process.

There are two, significant findings from our study of the three channel types, i.e., GluA2Q, GluA2Q/2R and GluA1/2R channels (Tables 1–4). We found R/G editing exerts its functional impact *on the flop*, but not flip, channel forms. In other words, the flip sequence alone seems to define the functional properties of their AMPA receptor channels (Tables 1–4). However, the flop sequence is coupled with the R/G editing to further expand channel functions. Second, by studying the GluA2Q/2R channel, we are able to keep the flip and flop sequences among all four subunits identical and thus determine whether the R/G editing status of the GluA2R isoform affects the GluA2Q/2R channel activities. For example, as compared with GluA2Q(G)/GluA2R(R) or simply GR (see Table 2), upon editing (or GG in Table 2), k_op_ and k_des_ are ~2-fold larger respectively. This same trend also holds true for the R/G unedited and edited pair of GluA1_flop_/GluA2R(R)_flop_ and GluA1_flop_/GluA2R(G)_flop_ heteromeric channels in terms of the k_op_ and k_des_ values (Tables 3 and 4). These results therefore suggest that R/G editing in GluA2R is functional in that R/G editing modulates the channel gating properties in a GluA2R-containing channel. This is noteworthy due to the fact that GluA2R has only been shown to form homomeric channels with femtosiemens conductance^40^. Unlike GluA2Q, expression of GluA2R alone in a heterologorous system, such as HEK-293 cells, does not generate appreciable whole-cell current response^58^.

Given the results from GluA2Q and GluA2Q/2R, it may not be surprising that the GluA2R flop variant, not the flip, also uses its R/G editing site to expand the function of the GluA1 flip and flop variants as the participating subunits in the GluA1/2R heteromeric channel configuration (Tables 3 and 4). There are several conclusions that we can draw from our data. (i) When GluA1_flip_/2R(R)_flop_ and GluA1_flop_/2R(R)_flop_ are compared, where the GluA2R is in the R/G *unedited* status, k_des_ and k_op_ in the former are 164 s^−1^ and 2.2 × 10^4^ s^−1^, but are 214 s^−1^ and 2.6 × 10^4^ s^−1^, respectively, for the latter channel form (Table 4). However, when both the GluA1 and GluA2R are in flop status, editing at the R/G site increases both k_des_ and k_op_ by ~2-fold (Table 4). Of note, the k_des_ and k_op_ for the GluA1 flop and GluA2R flop but R/G edited channel are as high as close to 400 s^−1^ and 5.6 × 10^4^ s^−1^ (Table 4). These results (Table 4) and the comparison indicate that the R/G editing is most effective in modulating the channel properties when both GluA1 and GluA2R are in the flop background, although minimally the GluA2R must be in the flop status (Tables 3 and 4). (ii) R/G editing increases P_op_ for GluA1/2R_flop_ channels (see the detail in Tables 3 and 4, including how P_op_ is estimated). The largest increase is seen in the R/G edited isoform or GluA1_flop_/2R(G)_flop_. P_op_ is the probability by which the channel can open once it is bound with glutamate^56^. Because R/G editing only leads to the increase of k_op_ while k_cl_ remains unaffected, the increase of P_op_ can be solely attributed to the increase of the rate of channel opening, upon binding of glutamate. A P_op_ of near unity suggests the channel-opening reaction, initiated by the binding of glutamate, is highly favorable.

The alternative way to see how the R/G editing in GluA2R affects the channel properties of the GluA1/2R is as follows. In the GluA2R R/G unedited group (Table 4), GluA1 flop exhibits ~1.2–1.3-fold larger value of both k_op_ and k_des_ than GluA1 flip, i.e., k_op_ is 2.6 × 10^4^ s^−1^ vs. 2.2 × 10^4^ s^−1^, and k_des_ is 214 s^−1^ vs. 164 s^−1^, respectively. However, in the GluA2R R/G edited group (Table 4), GluA1 flip and flop channels show a more significant difference in that (i) k_op_ of GluA1_flop_ is >2-fold larger than that of GluA1_flip_ (5.6 × 10^4^ s^−1^ vs. 2.0 × 10^4^ s^−1^) (Fig. 5b), and (ii) k_des_ goes up by another ~1.3-fold (398 s^−1^ vs. 308 s^−1^). In fact, k_des_ is increased by ~1.2–1.4-fold stepwise (Figs 4b and 5a). These results clearly demonstrate that the flip/flop module of GluA1 only realizes its functional divergence in the GluA1/2R_flop_ channel form. Precisely, the R/G editing in the GluA2R subunit (flop variant) is a specific regulatory mechanism to modulate the channel activity of GluA1 flip and flop variants in the rate of opening the channel, in response to the binding of glutamate, and then separately, the rate of channel desensitization on a slower time scale.

Several implications are apparent from our results. First, the GluA1 flip and flop splicing variants are indeed functionally different, but such a functional difference can only be realized in the GluA1/2R_flop_ channel form. A homomeric channel form is not a configuration where the flip and flop functional diversity of GluA1 can be expressed. A possible source of explanation (i.e., GluA1 homomeric channel vs. GluA1/2R heteromeric channel) is that GluA2R-containing channels exhibit an “O-shaped” structure as compared with an “N-shaped” structure for homomeric AMPA receptors^68,69^. Conceivably, the difference in the organization of these tetrameric assemblies could be the structural basis for the requirement of the GluA1/2R_flop_ background to expand the GluA1 flip and flop functional diversity. The fact that the GluA1/2R is a major native AMPA receptor population^13,16–18^ also makes this finding biologically relevant. Second, GluA2 exists nearly 100% in the R isoform (or the Q/R edited isoform) and GluA2R possesses an R/G editing site. As such, the R/G editing site on GluA2R is a unique molecular mechanism through which the function of the GluA1 flip and flop variants is expanded in the GluA1/2 heteromeric channel form. It is also conceivable that the R/G editing in the GluA2R_flop_ may be able to modulate the channel properties of other GluA2R-containing AMPA receptors, such as GluA2R_flop_/GluA3. Our finding that the R/G editing site on the GluA2R subunit can regulate the kinetic properties of the GluA1/2R heteromeric channels is also consistent with the hypothesis we have proposed earlier that GluA2R subunit plays a dominant role in shaping up the channel properties of GluA2R-containing channels^58^.

R/G editing in GluA2-4 is only differential throughout the brain and during development^33,38,47,48^. Furthermore, the R/G edited and unedited GluA2 isoforms in GluA2R (and even in GluA2Q) have differential kinetic properties, as we have shown from this study (Tables 1 and 2). The flip/flop splicing variants are also differentially expressed^26^. For example, in both hilar mossy cells and CA3 pyramidal neurons, the flip isoform is predominant in the GluA2 mRNAs^46^. In the medial nucleus of the trapezoid body relay neurons, only the flop isoforms of GluA1, GluA2, and GluA4 mRNAs are expressed^46^. In these neurons, the dominant expression of GluA4_flop_ and GluA2_flop_ mRNAs (55% and 24% of the total mRNAs, respectively) may be correlated to the fast and complete glutamate-induced desensitization^46^. Therefore, given that R/G isoforms and the flip/flop splicing variants generally co-exist, R/G editing in the flop background can be an important interplay in regulating AMPA receptor activities. Furthermore, R/G editing may be a unique, regulatory mechanism for the AMPA receptor subtype, since unlike Q/R editing, which exists in both AMPA and kainite receptors, R/G editing is exclusively found in AMPA receptors^70^.

As observed from our study, R/G editing in complex channel forms (either in GluA2Q/2R or GluA1/2R) has no effect on k_cl_ or the stability or the lifetime of the open channel. Yet, R/G editing increases the rate of channel opening. Because k_cl_ remains unaffected, the increase in k_op_ solely leads to the increase in P_op_. The increase in k_op_ suggests that the R/G editing isoform promotes a faster transition from the closed-channel to the open-channel state following the binding of glutamate to the receptor. It should be noted, however, that an increase in k_op_ cannot be understood at the discrete, as many as four, conductance levels^71–73^, due to the fact that our measurement and the subsequent determination of k_op_ is relevant only to the ensemble process. At the single channel level, the frequency of occurrence of these channels with different conductance is thought to depend on the number of subunits occupied by agonist^74,75^. Our study has also shown that R/G editing accelerates the entry of the channel into desensitizing state (Tables 1, 2 and 4) (note that channel desensitization is on a much slower time scale, as compared with channel opening). These results are consistent with the notion that channel desensitization starts from the closed-channel state of an AMPA receptor^10,57,76^, rather than open-channel state, and R/G editing therefore affects the closed-channel state of the receptor. In addition, the rate of channel desensitization, together with the channel closing and recovery rate, controls the excitability of the synapse to subsequent stimuli. For example, a rapidly gated AMPA channel could serve to decrease the rise time of the excitatory postsynaptic potential (EPSP)^77^ and to reduce the time interval between EPSP and initiation of postsynaptic action potential^46^. Therefore, R/G editing may play a role in these events.

It is interesting to note that the R/G editing site, which modulates the kinetic properties of the flop GluA2Q and GluA2R isoforms, is next to the flip/flop sequence cassette (Fig. 1b). The position of the R/G editing site in the amino acid sequence may be unique to its functional role and its relationship with the flop sequence. R/G editing and alternative splicing are known to be two coordinated processes^35^. Editing represses downstream intron splicing and affects downstream exon splicing by promoting flip over flop^78^. A study by Quirk *et al*. has revealed that two critical amino acid residues, i.e., Asn and Ala, that are right next to the R/G site in the flop sequence (Fig. 1a, right panel; Fig. 1b), contributes to splice variant-specific differences in the rate of channel desensitization^11^. That these two amino acid resides in the flop sequence region are different from the two residues at the equivalent positions in the flip sequence therefore suggests that R/G editing can be a unique, modulatory mechanism only for flop channels, and such a mechanism can be operative not just for the GluA2 but also possibly for GluA3 and GluA4 AMPA receptors. Structural studies show that these residues are located on helix J, which forms part of the intra-dimer interface of AMPA receptor ligand-binding cores, and that the stability of the interface regulates desensitization^68,79^. Complementary structure/function studies would be necessary to address how R/G editing site, from a structural point of view, is able to regulate AMPA receptor functions.

## Methods

### Cell Culture and Receptor Expression

cDNA plasmids encoding rat GluA2 AMPA receptors (edited at R/G site and unedited at Q/R site) and GluA1 in their respective flip and flop isoforms were used for transient expression^10,80^. GluA2 variants GluA2Q607R (edited at Q/R site) and GluA2G743R (unedited at R/G site) were created using QuikChange II site-directed mutagenesis kit (Agilent Technologies, CA) and corresponding mutations were confirmed by DNA sequencing. HEK-293S cells were cultured in the Dulbecco’s Modified Eagle’s Medium supplemented with 10% Fetal Bovine Serum, 100 U/mL penicillin, and 100 μg/mL streptomycin in a 37 °C, 6% CO_2_ humidified incubator. The cells were transiently transfected following a standard calcium phosphate transfection protocol^81^. Green fluorescent protein (GFP) was co-expressed as a marker for whole-cell recording. SV40 T Antigen (TAg) was also co-expressed for enhancing expression efficiency. The weight ratio of the plasmids for GluA2Q or GluA1, GFP, and TAg was 10:2:1 with GluA2 being 4–6 μg/35 mm dish. For GluA2Q/2R and GluA1/2R, the GluA2R was used 4-fold more than GluA2Q or GluA1 to ensure the formation of GluA2R-containing channels^58^. Cells were used for whole-cell recording from 24 hours after transfection.

### Whole-cell Recording and Laser-Pulse Photolysis

The whole-cell recordings and the laser-pulse photolysis measurement were performed at −60 mV and 22 °C. The recording electrode were made from borosilicate glass capillaries (World Precision Instruments, FL) that were filled with the intracellular buffer solution that contained 110 mM CsF, 30 mM CsCl, 4 mM NaCl, 0.5 mM CaCl_2_, 5 mM EGTA, and 10 mM HEPES (pH adjusted to 7.4 by CsOH). The extracellular buffer solution contains 150 mM NaCl, 3 mM KCl, 1 mM CaCl_2_, 1 mM MgCl_2_, and 10 mM HEPES (pH adjusted to 7.4 by NaOH). The series resistance during the recording was 2–3 MΩ and compensated to 40% on average. The whole cell capacitance was 1–2 pF. A glutamate solution was applied to the patched cell through a fast flow system consisting of a U-tube device^82^. The time resolution of this flow system measured using the rise time (10–90%) of the whole-cell current is 1.0 ± 0.2 ms^66^. Glutamate induced whole-cell current was recorded using an Axopatch-200B amplifier at a cutoff frequency of 2 kHz for fast flow measurement and 10–20 kHz for laser-pulse photolysis measurement by a built-in, four-pole Bessel filter, and digitized at 4 kHz −50 kHz sampling frequency, accordingly, using a digitizer Digidata 1322A (Molecular Devices, CA). Clampex 8 was used for data acquisition.

Laser-pulse photolysis technique was used for the measurements of the channel opening rate of AMPA receptors. The *γ*-*O*-(*α*-carboxy-2-nitrobenzyl)glutamate and 4-methoxy-7-nitroindolinyl (MNI)-caged-L-glutamate (Tocris, UK) were used for all laser-pulse measurements. Free or caged glutamate solutions were delivered to a cell through the same U-tube device as described in the last paragraph and our previous publication^80^. The cell was equilibrated with caged glutamate dissolved in extracellular buffer (up to 1 mM) for 250 ms and then irradiated by a laser pulse of 8 ns at 355 nm, produced by a Continuum Minilite II pulsed Q-switched Nd:YAG laser and tuned by a third harmonic generator. The laser beam was coupled into an optical fiber and delivered to the cell. The caged glutamate underwent photolysis upon the laser irradiation, releasing free glutamate with a t_1/2_ of ~30 μs^56^. It should be noted that the concentration of photolytically released glutamate was considered constant during the rise time in which the observed rate constant was measured^3^. In 1 ms time span, as in Fig. 2a, for instance, a glutamate molecule could have only diffused a root-mean-square distance of ~1.2 *µ*m, estimated by Fick’s second law; yet the laser irradiation area around a HEK-293 cell of ~10 *µ*m in diameter was 400–500 *µ*m. Thus, the diffusion of glutamate after photolysis, but within the period of our measurement of channel opening rate, was insignificant. In this estimate, we assumed the diffusion coefficient of glutamate was 7.5 × 10^–6^ cm^2^/s, based on the value for glutamine at room temperature^83^.

### Data analysis

Current amplitudes obtained in whole-cell recording were corrected for receptor desensitization during the rise time by a method previously described^82^. The necessity and procedures for this correction were explained previously^56^. In laser-pulse photolysis measurement of the channel opening rate of AMPA receptors in this study, >95% of the whole-cell current rising phase followed a single-exponential rate process for all measurable ligand concentrations, indicating the impact on these measurements by receptor desensitization was insignificant^10,56,66,80^. An observed channel opening rate constant, k_obs_, was determined by equation (1), where I_t_ represents the current amplitude at time t and I_A_ is the maximum current amplitude.

1 $${I}_{t}={I}_{A}(I-{e}^{-{k}_{obs}t})$$

By varying the concentration of glutamate, we collected a series of k_obs_. The concentration of each photolytically released free glutamate was determined, with the reference to the dose-response relationship, from at least two control whole-cell responses evoked by free glutamate solutions with known concentration. Using equation (2), we estimated k_op_ and k_cl_. Equation (2) was derived according to the general mechanism of channel opening shown in Fig. 1e; the definition of all the terms is in the legend. In deriving equation (2), we assumed that the rate of ligand binding is much faster than the rate of channel opening. The assumption was corroborated by the observation that more than 95% of the whole-cell current rising phase followed a single-exponential rate process^10,56,66,80^.

2 $${k}_{obs}={k}_{cl}+{k}_{op}{(\frac{L}{L+{K}_{1}})}^{n}$$

The dose-response relationship was analyzed in two ways. One was the Hill equation to obtain EC_50_ value, and the other was equation (3) to obtain the intrinsic equilibrium dissociation constant, K_1._ Equation (3) was also derived from the channel opening mechanism shown in Fig. 1e. In equation (3), I_A_ represents the normalized current amplitude, L the ligand concentration, I_M_ the current through per mole of receptor, R_M_ the number of moles of receptor embedded in cell membrane, Ф^−1^ the channel-opening equilibrium constant. The analysis of K_1_ was also correlated to the number of ligand, n.

3 $${I}_{A}={I}_{M}{R}_{M}\frac{{L}^{n}}{{L}^{n}+{\rm{\Phi }}{(L+{K}_{1})}^{n}}$$

Unless otherwise noted, three measurements from three cells were collected. Data analysis including linear and non-linear regression was performed using Origin 7. Standard deviations from the mean are reported unless otherwise noted. Two-tailed Welch’s t-tests were used for statistical comparisons of mean values between pairs assuming unequal variances. One-way ANOVA was used for multiple comparisons. Groups that have yielded significant* p* values (*p* ≤ 0.05) were followed by Tukey’s correction for full pairwise comparison (results are shown in corresponding figure legends). Differences with *p* ≤ 0.05 were considered significant.

## Electronic supplementary material

Supplementary Information
