# Supplementary material for: R/G editing in GluA2Rflop modulates the functional difference between GluA1 flip and flop variants in GluA1/2R heteromeric channels
Source: Sci Rep. 2017 Oct 20;7:13654. doi: 10.1038/s41598-017-13233-2 (PMC5651858; doi:10.1038/s41598-017-13233-2)
Supplement: Supplementary file 1 — Supplementary Information [file 41598_2017_13233_MOESM1_ESM.pdf]

R/G editing in GluA2R$_{flop}$ modulates the functional difference of GluA1 flip and flop variants in GluA1/2R heteromeric channels

Wei Wen[1], Chi-Yen Lin[1], and Li Niu[1]

[1]Department of Chemistry, and Center for Neuroscience Research, University at Albany, SUNY, Albany, New York 12222, United States. Correspondence and requests for materials should be addressed to L.N. (e-mail: lniu@albany.edu)

Supplementary Information

Table S1a
Non-linear Fitting of the Dose-Response Relationship for Homomeric GluA2Q R/G Channels When n = 1-4

(1) Non-linear regression of normalized dose-response data of GluA2Q channels at n = 1-4 by equation (3) (Methods). This is based on the fact that AMPA receptor is a tetramer and each subunit contains an agonist binding site. As such, n should be an integer ranging from 1 to 4.
(2) Because the dose-response curves between R and G sets were identical, within experimental errors (Supplementary Fig. S1a), we combined two sets of data or R/G data for fitting.

GluA2Q		n	K_1 (mM)	Φ	$I_M R_M$ (nA)	R^2
Flip	R/G	1	3.74±11.94	0.64±3.36	195±400	0.99
		2	1.20±0.16	0.51±0.20	150±34	0.96
		3	0.54±0.44	0.49±1.00	152±110	0.98
		4	0.36±0.26	0.57±1.16	161±128	0.98
Flop	R/G	1	3.54±12.19	0.90±5.93	240±746	0.98
		2	1.18±0.23	0.50±0.28	152±28	0.99
		3	0.60±0.21	0.58±0.55	172±64	0.99
		4	0.38±0.13	0.68±0.70	181±81	0.99

Table S1b
Non-linear Fitting of the Dose-Response Relationship for the Heteromeric GluA2Q/2R Channels When n = 1-4

(1) Non-linear regression of normalized dose-response data of GluA2Q/2R channels at n=1-4 by equation (3) (Methods).
(2) Because the dose-response curves between R and G sets were identical, within experimental errors (Supplementary Fig. S1b, c), we combined two sets of data (e.g., RR/GR data) for the non-linear fitting.

GluA2Q/2R		n	K_1 (mM)	Φ	$I_M R_M$ (nA)	R^2
Flip	RR/GR	1	2.59±0.33	0.51±0.12	160±82	0.98
		2	0.60±0.13	0.51±0.20	150±34	0.96
		3	0.52±0.16	0.50±0.14	152±54	0.94
		4	0.21±0.16	0.64±0.33	159±32	0.95
	RR/RG	1	1.37±0.58	1.42±0.83	264±75	0.99
		2	0.57±0.07	0.50±0.28	152±28	0.99
		3	0.57±0.40	0.50±0.26	152±79	0.98
		4	0.19±0.09	0.64±0.39	163±62	0.97
	RR/GG	1	2.01±0.49	0.87±0.35	206±62	0.99
		2	0.75±0.15	0.45±0.18	148±23	0.99
		3	0.38±0.23	0.59±0.12	162±79	0.98
		4	0.27±0.15	0.57±0.36	157±87	0.98
Flop	RR/GR	1	1.40±0.52	0.95±0.38	211±80	0.99
		2	0.51±0.13	0.51±0.24	155±23	0.99
		3	0.24±0.13	0.64±0.18	166±95	0.98
		4	0.16±0.08	0.66±0.34	164±98	0.98
	RR/RG	1	1.90±0.89	0.52±0.24	158±54	0.99
		2	0.50±0.09	0.53±0.13	150±26	0.97
		3	0.22±0.16	0.64±0.28	160±40	0.96
		4	0.14±0.09	0.72±0.46	166±43	0.96
	RR/GG	1	1.64±0.56	0.63±0.19	178±76	0.99
		2	0.50±0.18	0.50±0.13	156±16	0.99
		3	0.46±0.35	0.51±0.09	160±20	0.96
		4	0.18±0.07	0.58±0.42	163±74	0.98

Table S1c
Non-linear Fitting of the Dose-Response Relationship for the Heteromeric GluA1/2R Channels When n = 1-4

Non-linear regression of normalized dose-response data of GluA1/2R channels at n=1-4 by equation (3) (Methods).

GluA1/2R		n	K_1 (mM)	Φ	$I_M R_M$ (nA)	R^2
$A1_{flip}$	$2R(R)_{flip}$	1	1.27±0.23	1.37±0.85	250±67	0.99
		2	0.57±0.15	0.55±0.27	155±74	0.99
		3	0.29±0.17	0.65±0.28	165±21	0.99
		4	0.21±0.13	0.57±0.51	155±92	0.98
	$2R(G)_{flip}$	1	1.76±0.50	0.99±0.58	217±130	0.98
		2	0.60±0.17	0.54±0.34	155±58	0.99
		3	0.34±0.30	0.55±0.17	154±86	0.98
		4	0.20±0.15	0.65±0.34	161±44	0.98
	$2R(R)_{flop}$	1	3.23±0.67	0.47±0.91	157±76	0.99
		2	0.71±0.27	0.64±0.51	166±58	0.99
		3	0.43±0.38	0.57±0.25	156±96	0.98
		4	0.28±0.22	0.61±0.34	158±97	0.97
	$2R(G)_{flop}$	1	1.58±0.93	1.19±0.36	255±233	0.99
		2	0.72±0.35	0.46±0.35	162±51	0.99
		3	0.39±0.33	0.51±0.41	164±27	0.98
		4	0.23±0.18	0.70±0.48	185±64	0.98
$A1_{flop}$	$2R(R)_{flip}$	1	1.30±0.78	1.36±0.35	248±37	0.99
		2	0.55±0.46	0.53±0.25	152±99	0.99
		3	0.26±0.18	0.73±0.44	168±75	0.98
		4	0.17±0.12	0.64±0.27	157±75	0.98
	$2R(G)_{flip}$	1	1.96±0.60	0.98±0.80	208±30	0.99
		2	0.70±0.49	0.61±0.49	160±97	0.99
		3	0.43±0.31	0.53±0.89	150±91	0.99
		4	0.27±0.19	0.65±0.16	161±50	0.98
	$2R(R)_{flop}$	1	1.18±0.75	1.01±0.30	211±42	0.99
		2	0.50±0.17	0.50±0.22	150±75	0.99
		3	0.14±0.09	0.71±0.44	169±43	0.98
		4	0.28±0.22	0.61±0.34	158±97	0.97
	$2R(G)_{flop}$	1	2.03±0.52	0.75±0.68	188±78	0.99
		2	0.70±0.17	0.47±0.10	149±51	0.99
		3	0.32±0.25	0.70±0.47	170±31	0.98
		4	0.23±0.18	0.61±0.24	158±82	0.98

Table S2a

Linear Fitting of the Observed Channel Opening Rate Constant or k_{obs} as a Function of Glutamate Concentration for Homomeric GluA2Q R/G Channels when n = 1-4

(1) The k_{obs} data were fitted using equation (2) (Methods) with n varying from 1 to 4. In each of the linear regression, K_1 (n = 2) was used, and K_1 of 1.2 mM was used; the K_1 value was from the dose-response fitting shown in Supplementary Table 1a.

(2) Examples of these best fits are shown in Fig. 2b.

(3) The values (±SEM) of k_{op} and k_{cl} were yielded from fittings.

Receptor	R/G	n	k_{op} (x10^4 s^{-1})	k_{cl} (x10^3 s^{-1})	R^2
$GluA2Q_{flip}$	R	1	1.2±0.2	1.2±0.3	0.83
		2	5.8±0.5	1.5±0.1	0.95
		3	20.9±5.8	2.1±0.2	0.66
		4	95.4±32.1	2.3±0.2	0.60
	G	1	1.5±0.1	0.6±0.2	0.89
		2	5.5±0.4	1.5±0.1	0.94
		3	20.3±2.5	1.9±0.1	0.83
		4	87±12	2.1±0.1	0.79
$GluA2Q_{flop}$	R	1	1.8±0.1	0.9±0.2	0.93
		2	5.2±0.4	2.0±0.1	0.94
		3	29.0±2.0	2.4±0.1	0.94
		4	140.9±11.0	2.6±0.1	0.93
	G	1	2.0±0.2	1.9±0.3	0.90
		2	5.2±0.5	3.4±0.1	0.95
		3	18.1±2.4	4.1±0.2	0.83
		4	61.0±9.9	4.4±0.2	0.78

Table S2b

Linear Fitting of k_{obs} as a Function of Glutamate Concentration for GluA2Q/2R Channels when n = 1-4

(1) The k_{obs} data were fitted using equation (2) (Methods) with n varying from 1 to 4. In each of the linear regression, K_1 (n = 2) was used, and K_1 of 1.2 mM was used; the K_1 value was from the dose-response fitting in Supplementary Table S1b.

(2) An example of these best fits (n=2) are shown in Supplementary Fig.2, 3. The results are summarized in Table 2 for k_{op} and k_{cl} respectively.

(3) The values (±SEM) of k_{op} and k_{cl} were yielded from fittings.

Receptor	R/G	n	k_{op} (x10^4 s^{-1})	k_{cl} (x10^3 s^{-1})	R^2
$GluA2Q_{flip}/2R_{flip}$	RR	1	0.5±0.1	0.9±0.1	0.87
		2	1.2±0.1	1.3±0.1	0.90
		3	3.4±0.4	1.5±0.1	0.82
		4	10.0±1.3	1.5±0.1	0.77
	GR	1	0.5±0.1	0.4±0.2	0.80
		2	1.0±0.1	1.0±0.1	0.80
		3	1.7±0.2	1.4±0.1	0.78
		4	3.8±0.5	1.5±0.1	0.76
	RG	1	0.7±0.1	0.2±0.2	0.83
		2	1.3±0.2	1.1±0.1	0.85
		3	3.1±0.3	1.3±0.1	0.84
		4	7.5±0.9	1.5±0.1	0.83
	GG	1	0.6±0.1	0.7±0.1	0.90
		2	1.3±0.1	1.3±0.1	0.92
		3	3.3±0.2	1.5±0.1	0.89
		4	8.8±0.7	1.6±0.1	0.86
$GluA2Q_{flop}/2R_{flop}$	RR	1	0.8±0.1	1.2±0.1	0.91
		2	1.6±0.1	2.0±0.1	0.92
		3	3.9±0.3	2.3±0.1	0.89
		4	10.1±0.9	2.4±0.1	0.85
	GR	1	0.6±0.1	1.9±0.2	0.85
		2	0.9±0.1	2.7±0.1	0.86
		3	1.7±0.3	3.0±0.1	0.75
		4	3.3±0.6	3.0±0.1	0.70
	RG	1	1.5±0.1	0.3±0.3	0.89
		2	2.7±0.2	2.2±0.2	0.91
		3	6.0±0.5	2.8±0.1	0.89
		4	13.3±1.3	3.2±0.1	0.86
	GG	1	0.9±0.1	1.6±0.2	0.85
		2	1.7±0.2	2.6±0.2	0.86
		3	3.8±0.4	3.0±0.1	0.85
		4	9.1±1.1	3.2±0.1	0.83

Table S2c

Linear Fitting of k_{obs} as a Function of Glutamate Concentration for GluA1/2R Channels when n = 1-4

(4) The k_{obs} data were fitted using equation (2) (Methods) with n varying from 1 to 4. In each of the linear regression, K_1 (n = 2) was used, and K_1 of 1.2 mM was used; the K_1 value was from the dose-response fitting in Supplementary Table S1c.

(5) An example of these best fits (n=2) are shown in Fig. 4d. The results are summarized in Tables 3 and 4 for k_{op} and k_{cl} respectively.

(6) The values (±SEM) of k_{op} and k_{cl} were yielded from fittings.

Receptor		n	k_{op} ($x10^4$ s^{-1})	k_{cl} ($x10^3$ s^{-1})	R^2
$GluA1_{flip}$	$2R(R)_{flip}$	1	0.6±0.1	0.7±0.1	0.92
		2	1.5±0.1	1.3±0.1	0.93
		3	4.5±0.3	1.4±0.1	0.92
		4	13.7±0.9	1.6±0.1	0.90
	$2R(G)_{flip}$	1	0.6±0.1	0.8±0.1	0.89
		2	1.5±0.1	1.3±0.1	0.91
		3	4.7±0.4	1.5±0.1	0.89
		4	15.2±0.2	1.6±0.1	0.87
	$2R(R)_{flop}$	1	0.7±0.1	1.3±0.1	0.90
		2	2.2±0.1	1.9±0.1	0.90
		3	7.6±0.7	2.1±0.1	0.88
		4	27.5±0.3	2.2±0.1	0.85
	$2R(G)_{flop}$	1	0.8±0.1	1.3±0.1	0.90
		2	2.0±0.1	2.0±0.1	0.95
		3	5.8±0.3	2.3±0.1	0.95
		4	17.0±0.8	2.4±0.1	0.95
$GluA1_{flop}$	$2R(R)_{flip}$	1	0.8±0.1	0.9±0.1	0.94
		2	1.4±0.1	1.5±0.1	0.94
		3	4.3±0.3	1.6±0.1	0.91
		4	13.6±1.3	1.7±0.1	0.87
	$2R(G)_{flip}$	1	0.6±0.1	1.9±0.2	0.92
		2	2.1±0.1	1.6±0.1	0.93
		3	6.8±0.5	1.8±0.1	0.90
		4	22.6±1.9	1.9±0.1	0.88
	$2R(R)_{flop}$	1	1.2±0.1	0.4±0.1	0.94
		2	2.6±0.2	1.6±0.2	0.95
		3	6.9±0.4	2.1±0.1	0.93
		4	18.7±1.4	2.3±0.1	0.91
	$2R(G)_{flop}$	1	2.1±0.2	-0.1±0.4	0.87
		2	5.6±0.5	1.7±0.2	0.88
		3	18.6±1.7	2.3±0.2	0.87
		4	64.3±6.4	2.6±0.2	0.85

Table S3a

Nonlinear fitting with fixed k_{cl} for estimating k_{op}, K_1, and n using equation (2) (GluA2Q(R)$_{flip}$)

Initial values				outputs			
k_{cl}[a] (s^{-1})	k_{op} (s^{-1})	K_1 (mM)	n	k_{op} (s^{-1})	K_1 (mM)	n	R^2
1200	40000	1.0	2	42359	1.06	1.82	0.88
1200	45000	0.8	1	21283	0.96	1.47	0.9
1200	50000	1.2	3	61908	1.35	1.82	0.86
1200	55000	1.0	4	63176	1.98	1.97	0.81
1200	60000	0.5	2	41697	0.58	2.48	0.8
1500	40000	1.0	2	41092	1.02	1.95	0.89
1500	45000	0.8	1	40133	1.25	1.75	0.89
1500	50000	1.2	3	58759	1.33	1.90	0.89
1500	55000	1.0	4	62405	1.23	2.33	0.87
1500	60000	0.5	2	38024	0.61	2.49	0.88
1800	40000	1.0	2	42797	0.99	2.13	0.84
1800	45000	0.8	1	16078	1.01	1.55	0.75
1800	50000	1.2	3	59481	1.31	2.04	0.84
1800	55000	1.0	4	90521	1.09	2.04	0.85
1800	60000	0.5	2	36459	0.61	2.63	0.85

**Final outputs**	**k_{cl}[a] (s^{-1})**	**k_{op} (s^{-1})**	**K_1 (mM)**	**n**	**R^2**
	1200	46084	1.18	1.91	0.844
**Average**	1500	48082	1.08	2.08	0.886
	1800	49067	1.00	2.08	0.826

[a] Three fixed values of k_{cl} were chosen: 1200 s^{-1}, 1500 s^{-1}, and 1800 s^{-1}.

Table S3b

Nonlinear fitting with fixed k_{cl} and n for estimating k_{op} and K_1 using equation (2) (GluA2Q(R)$_{flip}$)

**Initial values**				**output**		
k_{cl}[a] (s^{-1})	k_{op} (s^{-1})	K_1 (mM)	n[b]	k_{op} (s^{-1})	K_1 (mM)	R^2
1200	40000	1.0	2	43000	0.90	0.85
1200	45000	0.8	2	40500	0.86	0.86
1200	50000	1.2	2	59062	1.07	0.85
1200	55000	1.0	2	54656	1.02	0.81
1200	60000	1.0	2	57000	1.05	0.85
1500	40000	1.0	2	41750	0.97	0.85
1500	45000	0.8	2	40162	0.95	0.89
1500	50000	1.2	2	52500	1.14	0.90
1500	55000	1.0	2	49809	1.10	0.89
1500	60000	1.0	2	53156	1.13	0.90
1800	40000	1.0	2	39900	1.10	0.82
1800	45000	0.8	2	34699	1.01	0.82
1800	50000	1.2	2	50000	1.26	0.83
1800	55000	1.0	2	45839	1.19	0.83
1800	60000	1.0	2	47250	1.21	0.83

**Final Outputs**	**k_{cl}[a] (s^{-1})**	**n[b]**	**k_{op} (s^{-1})**	**K_1 (mM)**	**R^2**
	1200	2	50843	0.98	0.84
**Ave.**	1500	2	47475	1.06	0.88
	1800	2	43537	1.15	0.82

[a] Three fixed values of k_{cl} were chosen: 1200 s^{-1}, 1500 s^{-1}, and 1800 s^{-1}.

[b] The fixed value of n is determined from the results in Supplementary Table S3a.

Table S3c

Nonlinear fitting with fixed k_{cl} for estimating k_{op}, K_1, and n using equation (2) ($GluA2Q(G)_{flip}$)

Initial values				outputs			
k_{cl}[a] (s^{-1})	k_{op} (s^{-1})	K_1 (mM)	n	k_{op} (s^{-1})	K_1 (mM)	n	R^2
1200	40000	1.0	2	41724	1.08	1.73	0.88
1200	45000	0.8	1	58699	1.00	1.43	0.91
1200	50000	1.2	3	69741	1.58	1.69	0.87
1200	55000	1.0	4	50739	1.08	2.46	0.86
1200	60000	0.5	2	44893	0.6	2.36	0.79
1500	40000	1.0	2	42092	1.01	1.90	0.88
1500	45000	0.8	1	23384	1.01	1.55	0.81
1500	50000	1.2	3	64309	1.36	1.87	0.88
1500	55000	1.0	4	54265	1.17	2.38	0.82
1500	60000	0.5	2	40464	0.59	2.44	0.86
1800	40000	1.0	2	40000	1.03	1.98	0.84
1800	45000	0.8	1	18891	1.01	1.54	0.79
1800	50000	1.2	3	76800	1.38	2.03	0.79
1800	55000	1.0	4	60206	1.08	2.46	0.84
1800	60000	0.5	2	37925	0.61	2.52	0.75

**Final outputs**	**k_{cl}[a] (s^{-1})**	**k_{op} (s^{-1})**	**K_1 (mM)**	**n**	**R^2**
	1200	53159	1.06	1.93	0.83
**Average**	1500	44902	1.02	2.03	0.85
	1800	46764	1.02	2.11	0.80

[a] Three fixed values of k_{cl} were chosen: 1200 s^{-1}, 1500 s^{-1}, and 1800 s^{-1}.

Table S3d

Nonlinear fitting with fixed k_{cl} and n for estimating k_{op} and K_1 using equation (2) ($GluA2Q(G)_{flip}$)

Initial values				output		
k_{cl}[a] (s^{-1})	k_{op} (s^{-1})	K_1 (mM)	n[b]	k_{op} (s^{-1})	K_1 (mM)	R^2
1200	40000	1.0	2	47348	0.86	0.84
1200	45000	0.8	2	45000	0.84	0.84
1200	50000	1.2	2	59375	1.02	0.83
1200	55000	1.0	2	57406	0.97	0.83
1200	60000	1.0	2	60000	1.00	0.83
1500	40000	1.0	2	53268	0.92	0.88
1500	45000	0.8	2	40500	0.86	0.88
1500	50000	1.2	2	56250	1.08	0.88
1500	55000	1.0	2	54306	1.06	0.88
1500	60000	1.0	2	57000	1.07	0.88
1800	40000	1.0	2	40000	1.00	0.80
1800	45000	0.8	2	38250	0.95	0.81
1800	50000	1.2	2	55125	1.20	0.84
1800	55000	1.0	2	49500	1.12	0.84
1800	60000	1.0	2	54534	1.18	0.84

**Final Outputs**	**k_{cl}[a] (s^{-1})**	**n[b]**	**k_{op} (s^{-1})**	**K_1 (mM)**	**R^2**
	1200	2	53826	0.94	0.83
**Ave.**	1500	2	52265	1.00	0.88
	1800	2	47482	1.09	0.82

[a] Three fixed values of k_{cl} were chosen: 1200 s^{-1}, 1500 s^{-1}, and 1800 s^{-1}.

[b] The fixed value of n is determined from the results in Supplementary Table S3c.

Table S3e

Nonlinear fitting with fixed k_{cl} for estimating k_{op}, K_1, and n using equation (2) (GluA2Q(R)$_{flop}$)

Initial values				outputs			
k_{cl} [a] (s^{-1})	k_{op} (s^{-1})	K_1 (mM)	n	k_{op} (s^{-1})	K_1 (mM)	n	R^2
1500	40000	1.0	2	43995	1.06	1.72	0.86
1500	45000	0.8	1	23188	0.98	1.79	0.78
1500	50000	1.2	3	66167	1.36	1.74	0.84
1500	55000	1.0	4	63267	1.10	2.46	0.82
1500	60000	0.5	2	44325	0.63	2.26	0.78
2000	40000	1.0	2	42222	0.97	1.93	0.88
2000	45000	0.8	1	43832	0.84	2.10	0.88
2000	50000	1.2	3	62500	1.30	1.90	0.89
2000	55000	1.0	4	64013	1.08	2.46	0.86
2000	60000	0.5	2	42771	0.60	2.48	0.88
2500	40000	1.0	2	40000	1.00	2.10	0.79
2500	45000	0.8	1	45240	0.84	2.34	0.81
2500	50000	1.2	3	59481	1.31	2.05	0.80
2500	55000	1.0	4	65749	1.11	2.48	0.84
2500	60000	0.5	2	34555	0.59	2.62	0.82

**Final outputs**	**k_{cl} [a] (s^{-1})**	**k_{op} (s^{-1})**	**K_1 (mM)**	**n**	**R^2**
**Average**	1500	48188	1.02	1.99	0.81
	2000	51067	0.95	2.17	0.87
	2500	49005	0.97	2.32	0.81

[a] Three fixed values of k_{cl} were chosen: 1500 s^{-1}, 2000 s^{-1}, and 2500 s^{-1}.

Table S3f

Nonlinear fitting with fixed k_{cl} and n for estimating k_{op} and K_1 using equation (2) (GluA2Q(R)$_{flop}$)

Initial values				output		
k_{cl}[a] (s^{-1})	k_{op} (s^{-1})	K_1 (mM)	n[b]	k_{op} (s^{-1})	K_1 (mM)	R^2
1500	40000	1.0	2	46250	0.80	0.82
1500	45000	0.8	2	45000	0.80	0.82
1500	50000	1.2	2	59765	0.95	0.81
1500	55000	1.0	2	57750	0.95	0.81
1500	60000	1.0	2	62250	0.98	0.8
2000	40000	1.0	2	45150	0.95	0.89
2000	45000	0.8	2	40500	0.98	0.88
2000	50000	1.2	2	50312	1.03	0.89
2000	55000	1.0	2	53719	1.05	0.89
2000	60000	1.0	2	55687	1.08	0.89
2500	40000	1.0	2	39900	1.10	0.78
2500	45000	0.8	2	34699	1.01	0.77
2500	50000	1.2	2	50000	1.26	0.79
2500	55000	1.0	2	45839	1.19	0.78
2500	60000	1.0	2	47250	1.21	0.78

**Final Outputs**	**k_{cl}[a] (s^{-1})**	**n[b]**	**k_{op} (s^{-1})**	**K_1 (mM)**	**R^2**
	1500	2	54203	0.90	0.81
**Ave.**	2000	2	49073	1.02	0.88
	2500	2	43537	1.15	0.78

[a] Three fixed values of k_{cl} were chosen: 1500 s^{-1}, 2000 s^{-1}, and 2500 s^{-1}..

[b] The fixed value of n is determined from the results in Supplementary Table S3e.

Table S3g

Nonlinear fitting with fixed k_{cl} for estimating k_{op}, K_1, and n using equation (2) $(GluA2Q(G)_{flop})$

Initial values				outputs			
k_{cl}[a] (s^{-1})	k_{op} (s^{-1})	K_1 (mM)	n	k_{op} (s^{-1})	K_1 (mM)	n	R^2
3000	40000	1.0	2	44712	1.21	1.75	0.77
3000	45000	0.8	1	19950	0.92	1.55	0.79
3000	50000	1.2	3	64156	1.23	2.00	0.80
3000	55000	1.0	4	79748	1.10	2.26	0.82
3000	60000	0.5	2	37200	0.62	2.37	0.71
3400	40000	1.0	2	40000	1.00	2.00	0.78
3400	45000	0.8	1	18310	0.70	1.77	0.78
3400	50000	1.2	3	62622	1.13	2.17	0.75
3400	55000	1.0	4	58413	1.24	2.03	0.77
3400	60000	0.5	2	35789	0.59	1.56	0.76
4000	40000	1.0	2	37333	1.06	2.13	0.72
4000	45000	0.8	1	12929	0.94	1.53	0.61
4000	50000	1.2	3	56141	0.99	2.48	0.74
4000	55000	1.0	4	72703	0.93	2.77	0.73
4000	60000	0.5	2	30777	0.61	2.71	0.74

**Final outputs**	**k_{cl}[a] (s^{-1})**	**k_{op} (s^{-1})**	**K_1 (mM)**	**n**	**R^2**
**Average**	1500	49153	1.01	1.99	0.77
	2000	43026	0.93	1.91	0.80
	2500	41976	0.90	2.32	0.70

[a] Three fixed values of k_{cl} were chosen: 3000 s^{-1}, 3400 s^{-1}, and 4000 s^{-1}.

Table S3h

Nonlinear fitting with fixed k_{cl} and n for estimating k_{op} and K_1 using equation (2) (GluA2Q(G)$_{flop}$)

Initial values				output		
k_{cl}[a] (s^{-1})	k_{op} (s^{-1})	K_1 (mM)	n[b]	k_{op} (s^{-1})	K_1 (mM)	R^2
3000	40000	1.0	2	42000	0.95	0.75
3000	45000	0.8	2	40500	0.90	0.75
3000	50000	1.2	2	53750	1.08	0.77
3000	55000	1.0	2	52593	1.07	0.76
3000	60000	1.0	2	54000	1.12	0.78
3400	40000	1.0	2	40000	1.00	0.78
3400	45000	0.8	2	35718	0.96	0.79
3400	50000	1.2	2	50000	1.20	0.77
3400	55000	1.0	2	48125	1.16	0.78
3400	60000	1.0	2	51000	1.19	0.78
4000	40000	1.0	2	36356	1.18	0.71
4000	45000	0.8	2	31893	1.08	0.71
4000	50000	1.2	2	45000	1.35	0.72
4000	55000	1.0	2	41387	1.27	0.72
4000	60000	1.0	2	42997	1.31	0.72

**Final Outputs**	**k_{cl}[a] (s^{-1})**	**n[b]**	**k_{op} (s^{-1})**	**K_1 (mM)**	**R^2**
	3000	2	48568	1.02	0.76
**Ave.**	3400	2	44968	1.10	0.78
	4000	2	39526	1.24	0.71

[a] Three fixed values of k_{cl} were chosen: 3000 s^{-1}, 3400 s^{-1}, and 4000 s^{-1}..

[b] The fixed value of n is determined from the results in Supplementary Table S3g.

Table S3i

Nonlinear fitting with fixed k_{cl} for estimating k_{op}, K_1, and n using equation (2) (GluA2Q_{flip}/2R_{flip})

	Initial values				output			
	k_{cl}[a] (s^{-1})	k_{op} (s^{-1})	K_1 (mM)	n	k_{op} (s^{-1})	K_1 (mM)	n	R^2
**RR**	1300	8000	0.5	2	8214	0.51	2.1	0.77
	1300	12000	0.6	1	4939	0.67	1.41	0.76
	1300	16000	1.0	3	20478	1.26	1.79	0.76
	1300	20000	0.4	4	22000	0.39	2.6	0.68
	1300	24000	0.6	2	17600	0.68	2.36	0.81
	1300	12000	0.6	2	10800	0.63	2.1	0.86
**RG**	1100	8000	0.5	2	8881	0.5	1.77	0.8
	1100	12000	0.6	1	13123	0.61	1.91	0.84
	1100	16000	1.0	3	22274	0.93	1.87	0.82
	1100	20000	0.4	4	24014	0.39	2.17	0.81
	1100	24000	0.6	2	21600	0.66	2.2	0.78
	1100	12000	0.6	2	12132	0.65	1.91	0.84
**GR**	1000	5000	0.5	2	6054	0.5	1.64	0.73
	1000	8000	0.6	1	8088	0.47	2.54	0.81
	1000	12000	1.0	3	15122	0.97	2.1	0.81
	1000	12000	0.4	4	13200	0.38	2.6	0.8
	1000	15000	0.6	2	11988	0.66	2.41	0.82
	1000	20000	0.6	2	13497	0.7	2.46	0.82
**GG**	1300	5000	0.5	2	6000	0.5	1.6	0.78
	1300	8000	0.6	1	9240	0.57	1.8	0.84
	1300	12000	1.0	3	16000	0.98	1.7	0.84
	1300	12000	0.4	4	15083	0.38	2.7	0.81
	1300	15000	0.6	2	14250	0.62	2.06	0.84
	1300	20000	0.6	2	18000	0.66	2.2	0.83

**Final Outputs**	**k_{cl}[a] (s^{-1})**	**k_{op} (s^{-1})**	**K_1 (mM)**	**n**	**R^2**
**RR**	1300	14005	0.69	2.06	0.77
**RG**	1100	17004	0.62	1.97	0.82
**GR**	1000	11325	0.61	2.29	0.80
**GG**	1300	13096	0.62	2.01	0.82

[a] Fixed values of k_{cl} were chosen for each combination. RR: 1300 s^{-1}, RG: 1100 s^{-1}, GR: 1000 s^{-1}, and GG: 1300 s^{-1}.

Table S3j

Nonlinear fitting with fixed k_{cl} and n for estimating k_{op} and K_1 using equation (2) ($GluA2Q_{flip}/2R_{flip}$)

	Initial values				output		
	k_{cl}[a] (s^{-1})	k_{op} (s^{-1})	K_1 (mM)	n[b]	k_{op} (s^{-1})	K_1 (mM)	R^2
**RR**	1300	8000	0.5	2	8000	0.5	0.81
	1300	12000	0.6	2	11400	0.65	0.81
	1300	16000	1.0	2	18000	0.9	0.82
	1300	20000	0.4	2	9933	0.6	0.81
	1300	24000	0.6	2	15356	0.78	0.81
	1300	12000	0.8	2	12900	0.72	0.82
**RG**	1100	8000	0.5	2	10185	0.4	0.79
	1100	12000	0.6	2	14204	0.52	0.8
	1100	16000	1.0	2	20918	0.68	0.8
	1100	20000	0.4	2	14043	0.51	0.8
	1100	24000	0.6	2	20250	0.68	0.8
	1100	12000	0.8	2	15900	0.56	0.8
**GR**	1000	5000	0.5	2	5937	0.43	0.75
	1000	8000	0.6	2	8400	0.57	0.76
	1000	12000	1.0	2	14250	0.85	0.83
	1000	12000	0.4	2	7500	0.52	0.8
	1000	15000	0.6	2	11250	0.73	0.84
	1000	20000	0.6	2	12500	0.78	0.83
**GG**	1300	5000	0.5	2	6298	0.36	0.84
	1300	8000	0.6	2	9975	0.51	0.85
	1300	12000	1.0	2	15937	0.71	0.84
	1300	12000	0.4	2	9000	0.49	0.84
	1300	15000	0.6	2	13500	0.65	0.84
	1300	20000	0.6	2	16125	0.72	0.84

**Final Outputs**	**k_{cl}[a] (s^{-1})**	**k_{op} (s^{-1})**	**K_1 (mM)**	**n[b]**	**R^2**
**RR**	1300	12598	0.69	2.00	0.81
**RG**	1100	15917	0.56	2.00	0.80
**GR**	1000	9973	0.65	2.00	0.80
**GG**	1300	11806	0.57	2.00	0.84

[a] Fixed values of k_{cl} were chosen for each combination. RR: 1300 s^{-1}, RG: 1100 s^{-1}, GR: 1000 s^{-1}, and GG: 1300 s^{-1}.

[b] The fixed value of n is determined from the results in Supplementary Table S3i.

Table S3k

Nonlinear fitting with fixed k_{cl} for estimating k_{op}, K_1, and n using equation (2) (GluA2Q_{flop}/2R_{flop})

	Initial values				output			
	k_{cl}[a] (s^{-1})	k_{op} (s^{-1})	K_1 (mM)	n	k_{op} (s^{-1})	K_1 (mM)	n	R^2
**RR**	2000	8000	0.5	2	9600	0.5	1.6	0.84
	2000	12000	0.6	1	11522	0.63	1.73	0.84
	2000	16000	1.0	3	27789	1.09	1.64	0.84
	2000	20000	0.4	4	26507	0.43	2.68	0.8
	2000	24000	0.6	2	22230	0.64	2.02	0.84
	2000	12000	0.6	2	14280	0.6	1.73	0.85
**RG**	2200	15000	0.5	2	14547	0.53	1.81	0.84
	2200	18000	0.6	1	14140	0.67	2.23	0.75
	2200	21000	1.0	3	48413	1.09	1.65	0.84
	2200	24000	0.4	4	30723	0.38	2.43	0.83
	2200	27000	0.6	2	29377	0.61	1.81	0.84
	2200	30000	0.6	2	31477	0.61	1.9	0.84
**GR**	2700	6000	0.5	2	6720	0.49	1.87	0.77
	2700	9000	0.6	1	6466	0.67	1.85	0.71
	2700	12000	1.0	3	16125	0.98	1.64	0.74
	2700	15000	0.4	4	17697	0.4	2.01	0.74
	2700	18000	0.6	2	16200	0.66	2.1	0.73
	2700	21000	0.6	2	18200	0.63	2.27	0.77
**GG**	2600	12000	0.5	2	13038	0.5	1.81	0.74
	2600	15000	0.6	1	8666	0.65	1.76	0.7
	2600	18000	1.0	3	26904	0.94	1.74	0.74
	2600	21000	0.4	4	25796	0.4	2.86	0.7
	2600	24000	0.6	2	22800	0.62	2.07	0.74
	2600	27000	0.6	2	24300	0.63	2.1	0.73

**Final Outputs**	**k_{cl}[a] (s^{-1})**	**k_{op} (s^{-1})**	**K_1 (mM)**	**n**	**R^2**
**RR**	2000	18655	0.65	1.90	0.84
**RG**	2200	28113	0.65	1.97	0.82
**GR**	2700	13568	0.64	1.96	0.74
**GG**	2600	20251	0.62	2.06	0.73

[a] Fixed values of k_{cl} were chosen for each combination. RR: 2000 s^{-1}, RG: 2200 s^{-1}, GR: 2700 s^{-1}, and GG: 2600 s^{-1}.

Table S3l

Nonlinear fitting with fixed k_{cl} and n for estimating k_{op} and K_1 using equation (2) ($GluA2Q_{flop}/2R_{flop}$)

	Initial values				output		
	k_{cl}[a] (s^{-1})	k_{op} (s^{-1})	K_1 (mM)	n[b]	k_{op} (s^{-1})	K_1 (mM)	R^2
**RR**	2000	8000	0.5	2	10221	0.36	0.85
	2000	12000	0.6	2	14761	0.48	0.85
	2000	16000	1.0	2	20700	0.63	0.84
	2000	20000	0.4	2	15000	0.49	0.85
	2000	24000	0.6	2	21600	0.65	0.84
	2000	12000	0.8	2	15525	0.5	0.85
**RG**	2200	15000	0.5	2	19089	0.37	0.84
	2200	18000	0.6	2	23809	0.43	0.84
	2200	21000	1.0	2	29695	0.52	0.84
	2200	24000	0.4	2	22800	0.42	0.84
	2200	27000	0.6	2	30375	0.54	0.84
	2200	30000	0.8	2	36375	0.6	0.84
**GR**	2700	6000	0.5	2	7558	0.36	0.73
	2700	9000	0.6	2	10420	0.5	0.76
	2700	12000	1.0	2	15339	0.69	0.76
	2700	15000	0.4	2	10929	0.52	0.75
	2700	18000	0.6	2	15581	0.67	0.73
	2700	21000	0.6	2	16391	0.72	0.75
**GG**	2600	12000	0.5	2	13500	0.45	0.74
	2600	15000	0.6	2	16687	0.53	0.74
	2600	18000	1.0	2	24653	0.69	0.74
	2600	21000	0.4	2	15421	0.51	0.74
	2600	24000	0.6	2	21600	0.65	0.74
	2600	27000	0.6	2	23930	0.68	0.74

**Final Outputs**	**k_{cl}[a] (s^{-1})**	**k_{op} (s^{-1})**	**K_1 (mM)**	**n[b]**	**R^2**
**RR**	2000	16301	0.52	2.00	0.85
**RG**	2200	27024	0.48	2.00	0.84
**GR**	2700	12703	0.58	2.00	0.75
**GG**	2600	19299	0.59	2.00	0.74

[a] Fixed values of k_{cl} were chosen for each combination. RR: 2000 s^{-1}, RG: 2200 s^{-1}, GR: 2700 s^{-1}, and GG: 2600 s^{-1}.

[b] The fixed value of n is determined from the results in Supplementary Table S3k.

Table S3m

Nonlinear fitting with fixed k_{cl} for estimating k_{op}, K_1, and n using equation (2) (GluA1$_{flip}$/2R)

	Initial values				output			
	k_{cl}[a] (s^{-1})	k_{op} (s^{-1})	K_1 (mM)	n	k_{op} (s^{-1})	K_1 (mM)	n	R^2
**2R(R)$_{flip}$**	1300	8000	0.5	2	12260	0.51	1.97	0.87
	1300	12000	0.6	1	7568	0.72	1.33	0.8
	1300	16000	1.0	3	27403	1.14	1.72	0.87
	1300	20000	0.4	4	24767	0.4	2.85	0.83
	1300	24000	0.6	2	21788	0.65	2.12	0.87
	1300	12000	0.6	2	21514	0.76	1.96	0.87
**2R(G)$_{flip}$**	1300	8000	0.5	2	8800	0.48	1.8	0.81
	1300	12000	0.6	1	24602	1.06	1.72	0.82
	1300	16000	1.0	3	27279	1.15	1.73	0.82
	1300	20000	0.4	4	24357	0.39	2.9	0.75
	1300	24000	0.6	2	21625	0.63	2.14	0.81
	1300	12000	0.6	2	12800	0.59	1.87	0.82
**2R(R)$_{flop}$**	1900	14000	0.5	2	12600	0.53	2.1	0.82
	1900	17000	0.6	1	7033	0.71	1.38	0.77
	1900	20000	1.0	3	25166	1.09	1.82	0.81
	1900	23000	0.4	4	26721	0.4	2.33	0.77
	1900	27000	0.6	2	22937	0.65	2.27	0.81
	1900	20000	0.6	2	18049	0.63	2.13	0.81
**2R(G)$_{flop}$**	2000	16000	0.5	2	28689	0.63	2.22	0.9
	2000	19000	0.6	1	13341	0.76	1.47	0.85
	2000	22000	1.0	3	37893	1.2	1.74	0.9
	2000	25000	0.4	4	29631	0.42	2.8	0.88
	2000	28000	0.6	2	26133	0.64	2.13	0.9
	2000	22000	0.6	2	22273	0.63	2.03	0.89

**Final Outputs**	**k_{cl}[a] (s^{-1})**	**k_{op} (s^{-1})**	**K_1 (mM)**	**n**	**R^2**
**2R(R)$_{flip}$**	1300	19217	0.70	1.99	0.85
**2R(G)$_{flip}$**	1300	19911	0.72	2.03	0.81
**2R(R)$_{flop}$**	1900	18751	0.67	2.01	0.80
**2R(G)$_{flop}$**	2000	26327	0.71	2.07	0.89

[a] Fixed values of k_{cl} were chosen for each combination. 2R(R)$_{flip}$: 1300 s^{-1}, 2R(G)$_{flip}$: 1300 s^{-1}, 2R(R)$_{flop}$: 1900 s^{-1}, and 2R(G)$_{flop}$: 2000 s^{-1}.

Table S3n

Nonlinear fitting with fixed k_{cl} and n for estimating k_{op} and K_1 using equation (2) ($GluA1_{flip}$/2R)

	Initial values				output		
	k_{cl}[a] (s^{-1})	k_{op} (s^{-1})	K_1 (mM)	n[b]	k_{op} (s^{-1})	K_1 (mM)	R^2
**$2R(R)_{flip}$**	1300	8000	0.5	2	10185	0.43	0.86
	1300	12000	0.6	2	14175	0.54	0.87
	1300	16000	1.0	2	20627	0.7	0.87
	1300	20000	0.4	2	13500	0.52	0.88
	1300	24000	0.6	2	20400	0.68	0.87
	1300	12000	0.8	2	15185	0.57	0.87
**$2R(G)_{flip}$**	1300	8000	0.5	2	9450	0.41	0.82
	1300	12000	0.6	2	13500	0.54	0.82
	1300	16000	1.0	2	21249	0.72	0.81
	1300	20000	0.4	2	13125	0.52	0.82
	1300	24000	0.6	2	20400	0.71	0.81
	1300	12000	0.8	2	15332	0.58	0.82
**$2R(R)_{flop}$**	1900	14000	0.5	2	12600	0.56	0.82
	1900	17000	0.6	2	15300	0.65	0.82
	1900	20000	1.0	2	23750	0.85	0.81
	1900	23000	0.4	2	12678	0.56	0.82
	1900	27000	0.6	2	19532	0.76	0.82
	1900	20000	0.8	2	21000	0.8	0.81
**$2R(G)_{flop}$**	2000	16000	0.5	2	16731	0.51	0.89
	2000	19000	0.6	2	19950	0.6	0.89
	2000	22000	1.0	2	27998	0.73	0.9
	2000	25000	0.4	2	16785	0.52	0.89
	2000	28000	0.6	2	24806	0.68	0.89
	2000	22000	0.8	2	25458	0.71	0.9

**Final Outputs**	**k_{cl}[a] (s^{-1})**	**k_{op} (s^{-1})**	**K_1 (mM)**	**n[b]**	**R^2**
**$2R(R)_{flip}$**	1300	15679	0.57	2.00	0.87
**$2R(G)_{flip}$**	1300	15509	0.58	2.00	0.82
**$2R(R)_{flop}$**	1900	17477	0.70	2.00	0.82
**$2R(G)_{flop}$**	2000	21955	0.63	2.00	0.89

[a] Fixed values of k_{cl} were chosen for each combination. $2R(R)_{flip}$: 1300 s^{-1}, $2R(G)_{flip}$: 1300 s^{-1}, $2R(R)_{flop}$: 1900 s^{-1}, and $2R(G)_{flop}$: 2000 s^{-1}.

[b] The fixed value of n is determined from the results in Supplementary Table S3m.

Table S3o

Nonlinear fitting with fixed k_{cl} for estimating k_{op}, K_1, and n using equation (2) (GluA1$_{flop}$/2R)

	Initial values				output			
	k_{cl}[a] (s^{-1})	k_{op} (s^{-1})	K_1 (mM)	n	k_{op} (s^{-1})	K_1 (mM)	n	R^2
**2R(R)$_{flip}$**	1500	8000	0.5	2	9223	0.5	1.81	0.88
	1500	11000	0.6	1	11080	0.8	1.56	0.87
	1500	14000	1.0	3	24329	1.15	1.71	0.88
	1500	17000	0.4	4	21615	0.4	2.79	0.84
	1500	20000	0.6	2	18458	0.63	2.09	0.88
	1500	14000	0.6	2	15006	0.6	1.98	0.88
**2R(G)$_{flip}$**	1600	15000	0.5	2	14250	0.52	2.07	0.85
	1600	18000	0.6	1	9000	0.78	1.37	0.79
	1600	21000	1.0	3	33443	1.2	1.78	0.85
	1600	24000	0.4	4	28505	0.4	3.04	0.81
	1600	27000	0.6	2	25515	0.66	2.2	0.85
	1600	21000	0.6	2	21315	0.63	2.11	0.85
**2R(R)$_{flop}$**	1600	20000	0.5	2	21825	0.51	1.82	0.9
	1600	23000	0.6	1	27301	0.8	1.58	0.9
	1600	26000	1.0	3	49089	1.1	1.65	0.9
	1600	29000	0.4	4	36659	0.4	2.57	0.87
	1600	32000	0.6	2	33006	0.62	1.9	0.9
	1600	26000	0.6	2	28415	0.63	1.82	0.9
**2R(G)$_{flop}$**	1700	45000	0.5	2	40500	0.53	2.1	0.78
	1700	50000	0.6	1	26250	0.75	1.45	0.74
	1700	56000	1.0	3	84654	1.18	1.77	0.78
	1700	60000	0.4	4	73372	0.44	2.85	0.75
	1700	65000	0.6	2	63429	0.65	2.16	0.78
	1700	56000	0.6	2	58339	0.64	2.12	0.78

**Final Outputs**	**k_{cl}[a] (s^{-1})**	**k_{op} (s^{-1})**	**K_1 (mM)**	**n**	**R^2**
**2R(R)$_{flip}$**	1500	16619	0.68	1.99	0.87
**2R(G)$_{flip}$**	1600	22005	0.70	2.10	0.83
**2R(R)$_{flop}$**	1600	32716	0.68	1.89	0.90
**2R(G)$_{flop}$**	1700	57757	0.70	2.08	0.77

[a] Fixed values of k_{cl} were chosen for each combination. 2R(R)$_{flip}$: 1500 s^{-1}, 2R(G)$_{flip}$: 1600 s^{-1}, 2R(R)$_{flop}$: 1600 s^{-1}, and 2R(G)$_{flop}$: 1700 s^{-1}.

Table S3p

Nonlinear fitting with fixed k_{cl} and n for estimating k_{op} and K_1 using equation (2) ($GluA1_{flop}$/2R)

	Initial values				output		
	k_{cl}[a] (s^{-1})	k_{op} (s^{-1})	K_1 (mM)	n[b]	k_{op} (s^{-1})	K_1 (mM)	R^2
**$2R(R)_{flip}$**	1500	8000	0.5	2	9500	0.43	0.88
	1500	11000	0.6	2	12993	0.53	0.88
	1500	14000	1.0	2	19175	0.7	0.88
	1500	17000	0.4	2	12048	0.51	0.88
	1500	20000	0.6	2	18000	0.68	0.88
	1500	14000	0.8	2	16734	0.64	0.88
**$2R(G)_{flip}$**	1600	15000	0.5	2	14250	0.55	0.85
	1600	18000	0.6	2	18000	0.63	0.85
	1600	21000	1.0	2	25675	0.8	0.85
	1600	24000	0.4	2	13968	0.53	0.85
	1600	27000	0.6	2	22950	0.75	0.85
	1600	21000	0.8	2	23703	0.76	0.85
**$2R(R)_{flop}$**	1600	20000	0.5	2	23635	0.45	0.9
	1600	23000	0.6	2	27312	0.51	0.9
	1600	26000	1.0	2	35333	0.6	0.9
	1600	29000	0.4	2	24468	0.46	0.9
	1600	32000	0.6	2	33600	0.57	0.9
	1600	26000	0.8	2	33462	0.58	0.9
**$2R(G)_{flop}$**	1700	45000	0.5	2	40500	0.56	0.78
	1700	50000	0.6	2	47500	0.63	0.78
	1700	56000	1.0	2	71777	0.81	0.78
	1700	60000	0.4	2	38390	0.54	0.78
	1700	65000	0.6	2	55250	0.68	0.78
	1700	56000	0.8	2	60200	0.72	0.78

**Final Outputs**	**k_{cl}[a] (s^{-1})**	**k_{op} (s^{-1})**	**K_1 (mM)**	**n[b]**	**R^2**
**$2R(R)_{flip}$**	1500	14742	0.58	2.00	0.88
**$2R(G)_{flip}$**	1600	19758	0.67	2.00	0.85
**$2R(R)_{flop}$**	1600	29635	0.53	2.00	0.90
**$2R(G)_{flop}$**	1700	52270	0.66	2.00	0.78

Fixed values of k_{cl} were chosen for each combination. $2R(R)_{flip}$: 1500 s^{-1}, $2R(G)_{flip}$: 1600 s^{-1}, $2R(R)_{flop}$: 1600 s^{-1}, and $2R(G)_{flop}$: 1700 s^{-1}.

[b] The fixed value of n is determined from the results in Supplementary Table S3o.

Table S4a
Non-linear Fitting of k_{obs} with a Fixed k_{cl} Value to Estimate k_{op} and n for Homomeric GluA2Q channels

To achieve a better estimate of n and k_{op} by non-linear regression (Simplex algorithm) using equation (2), the values of k_{cl} were fixed. According to equation (2), $k_{obs} \approx k_{cl}$ when $L \ll K_1$, suggesting that (a) k_{obs} at a low glutamate concentration would reflect k_{cl} and (b) the value of k_{cl} is independent variable. Based on this rationale, we identified a k_{obs} determined at ~4% fraction of the open channel to be the k_{cl} for each channel combination (e.g. k_{cl} = 1500 s^{-1} for GluA2Q(G)$_{flip}$). In addition, K_1 value was also fixed at 1.2 mM. This value was obtained from analysis of the dose-response data as in Supplementary Table S1a.

Receptor	k_{cl} (s^{-1})	k_{op} (s^{-1})	n	R^2
**GluA2Q(R)$_{flip}$**	$1.5x10^3$	$(6.0±2.3)x10^4$	1.95±0.48	0.90
**GluA2Q(G)$_{flip}$**	$1.5x10^3$	$(5.8±2.9)x10^4$	2.00±0.30	0.79
**GluA2Q(R)$_{flop}$**	$2.0x10^3$	$(5.5±1.7)x10^4$	1.93±0.17	0.89
**GluA2Q(G)$_{flop}$**	$3.4x10^3$	$(5.6±1.9)x10^4$	1.96±0.21	0.87

Table S4b

Non-linear Fitting of k_{obs} with a Fixed k_{cl} Value to Estimate k_{op} and n for Heteromeric GluA2Q/2R channels with Different R/G Editing Status in Participating Subunits

To achieve a better estimate of n and k_{op} by non-linear regression using equation (2), the values of k_{cl} were fixed. According to equation (2), $k_{obs} \approx k_{cl}$ when $L \ll K_1$, suggesting that (a) k_{obs} at a low glutamate concentration would reflect k_{cl} and (b) the value of k_{cl} is independent of the n value. Based on this rationale, we identified a k_{obs} determined at ~4% fraction of the open channel to be the k_{cl} for each channel combination (e.g. k_{cl} = 1200 s^{-1} for GluA2Q(R)$_{flip}$/2R(R)$_{flip}$). In addition, K_1 value was also fixed at the corresponding values as shown in Supplementary Table S1b for n = 2.

Receptor	k_{cl}	k_{op} (s^{-1})	n	R^2
**GluA2Q(R)$_{flip}$/2R(R)$_{flip}$**	1.2×10^3	$(1.2 \pm 0.4) \times 10^4$	2.00±0.23	0.86
**GluA2Q(G)$_{flip}$/2R(R)$_{flip}$**	1.0×10^3	$(1.1 \pm 0.3) \times 10^4$	1.96±0.25	0.81
**GluA2Q(R)$_{flip}$/2R(G)$_{flip}$**	1.1×10^3	$(1.4 \pm 0.5) \times 10^4$	2.26±0.35	0.89
**GluA2Q(G)$_{flip}$/2R(G)$_{flip}$**	1.3×10^3	$(1.4 \pm 0.3) \times 10^4$	2.00±0.17	0.84
**GluA2Q(R)$_{flop}$/2R(R)$_{flop}$**	2.0×10^3	$(1.6 \pm 0.3) \times 10^4$	2.00±0.17	0.85
**GluA2Q(G)$_{flop}$/2R(R)$_{flop}$**	2.7×10^3	$(0.9 \pm 0.3) \times 10^4$	2.00±0.37	0.85
**GluA2Q(R)$_{flop}$/2R(G)$_{flop}$**	2.2×10^3	$(3.0 \pm 0.5) \times 10^4$	1.96±0.15	0.83
**GluA2Q(G)$_{flop}$/2R(G)$_{flop}$**	2.6×10^3	$(1.7 \pm 0.5) \times 10^4$	2.00±0.25	0.84

Table S4c

Non-linear Fitting of k_{obs} with a Fixed k_{cl} Value to Estimate k_{op} and n for Heteromeric GluA1/2R channels with Different R/G Editing Status in Participating Subunits

To achieve a better estimate of n and k_{op} by non-linear regression using equation (2), the values of k_{cl} were fixed. According to equation (2), $k_{obs} \approx k_{cl}$ when $L \ll K_1$, suggesting that (a) k_{obs} at a low glutamate concentration would reflect k_{cl} and (b) the value of k_{cl} is independent of the n value. Based on this rationale, we identified a k_{obs} determined at ~4% fraction of the open channel to be the k_{cl} for each channel combination (e.g. k_{cl} = 1300 s^{-1} for $GluA1_{flip}/2R(R)_{flip}$). In addition, K_1 value was also fixed at the corresponding values as shown in Supplementary Table S1c for n = 2.

**Receptor**	**k_{cl}**	**k_{op} (s^{-1})**	**n**	**R^2**
**$GluA1_{flip}/2R(R)_{flip}$**	$1.3x10^3$	$(1.5±0.3)x10^4$	2.00±0.14	0.87
**$GluA1_{flip}/2R(G)_{flip}$**	$1.3x10^3$	$(1.6±0.4)\ x10^4$	2.00±0.18	0.82
**$GluA1_{flip}/2R(R)_{flop}$**	$1.9x10^3$	$(2.0±0.7)\ x10^4$	2.10±0.26	0.81
**$GluA1_{flip}/2R(G)_{flop}$**	$2.0x10^3$	$(2.2±0.4)\ x10^4$	1.85±0.13	0.89
**$GluA1_{flop}/2R(R)_{flip}$**	$1.5x10^3$	$(1.4±0.4)x10^4$	2.06±0.20	0.88
**$GluA1_{flop}/2R(G)_{flip}$**	$1.6x10^3$	$(2.0±0.5)\ x10^4$	1.99±0.18	0.85
**$GluA1_{flop}/2R(R)_{flop}$**	$1.6x10^3$	$(2.7±0.4)\ x10^4$	2.00±0.13	0.90
**$GluA1_{flop}/2R(G)_{flop}$**	$1.7x10^3$	$(5.6±1.8)\ x10^4$	2.00±0.22	0.89

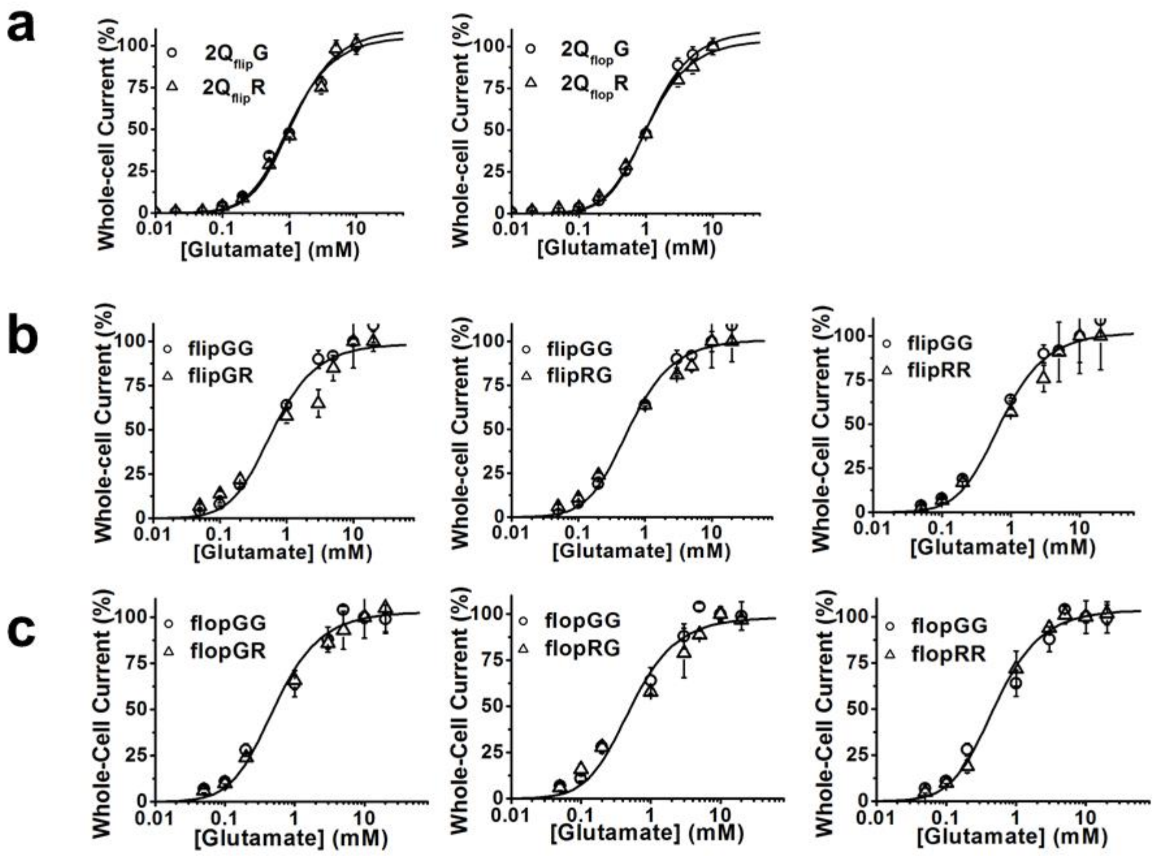

Figure S1. Effects of R/G editing on the dose-response relationship of homomeric and complex GluA2 channels. (a) Superimposition of the dose-response curves of for GluA2Q_{flip} (left) and GluA2Q_{flop} (right) channels edited (G, ○) and unedited (R, Δ) at R/G site. These fittings were produced at n = 2. Table S1 shows the fitted results when n was varied. **(b)** Non-linear fits of the combined dose-response data for GluA2Q_{flip}/2R_{flip} complex channels, shown as each of the inset labels. **(c)** Non-linear fits of the combined dose-response data for GluA2Q_{flop}/2R_{flop} complex channels, shown as each of the inset labels.

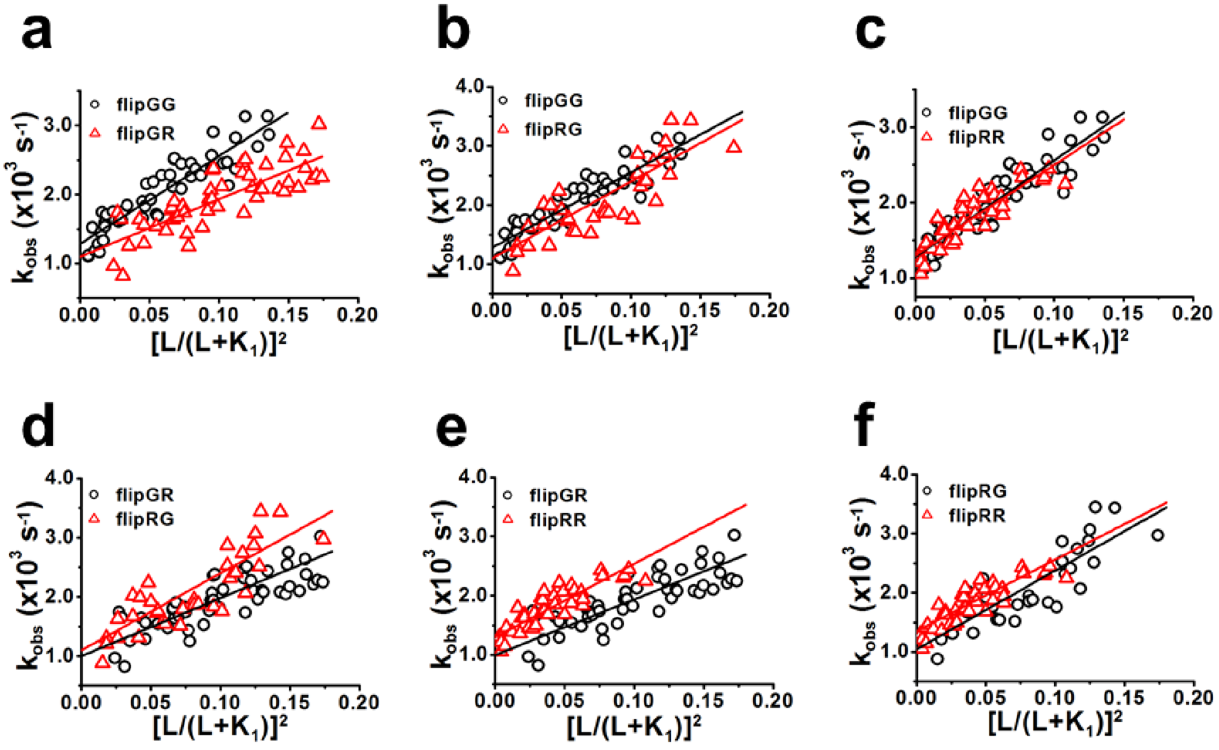

Figure S2. Linear fitting of k_{obs} as a function of glutamate concentration for GluA2Q$_{flip}$/2R$_{flip}$ channels at n=2. Channels with different R/G editing degrees are plotted in pairs for comparison.

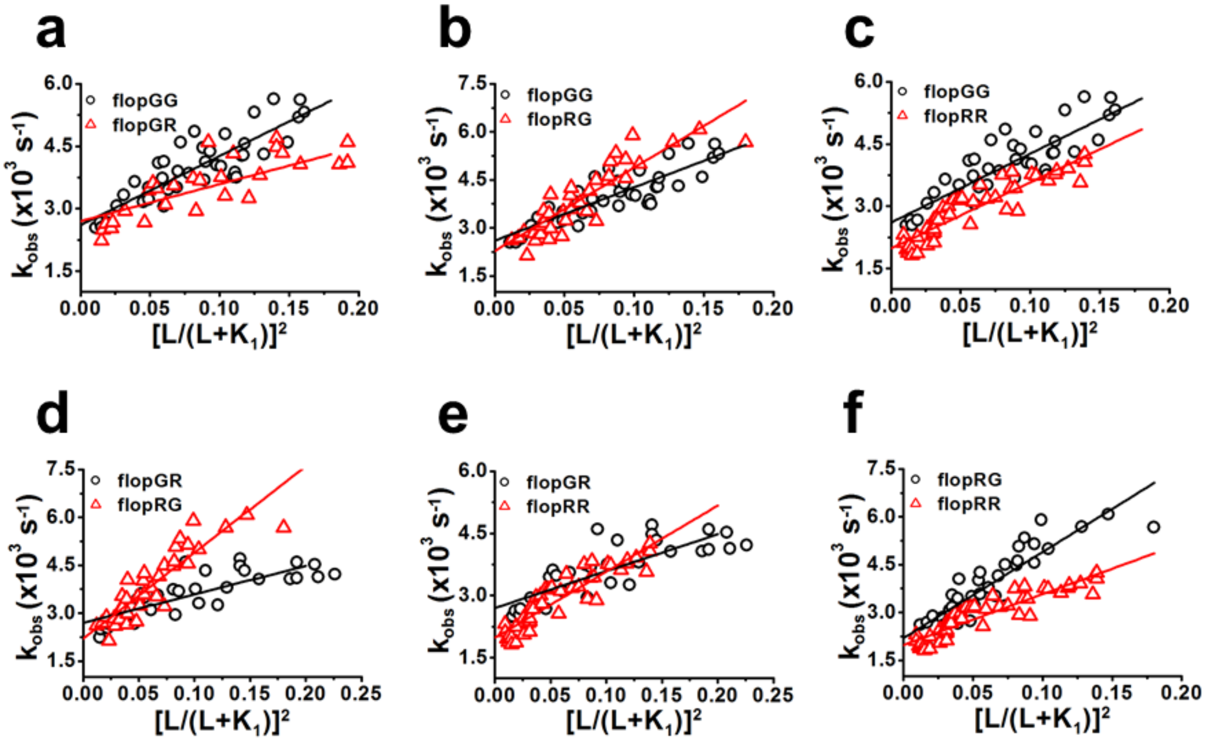

Figure S3. Linear fitting of k_{obs} as a function of glutamate concentration for GluA2Q$_{flop}$/2R$_{flop}$ channels at n=2. Channels with different R/G editing degrees are plotted in pairs for comparison.

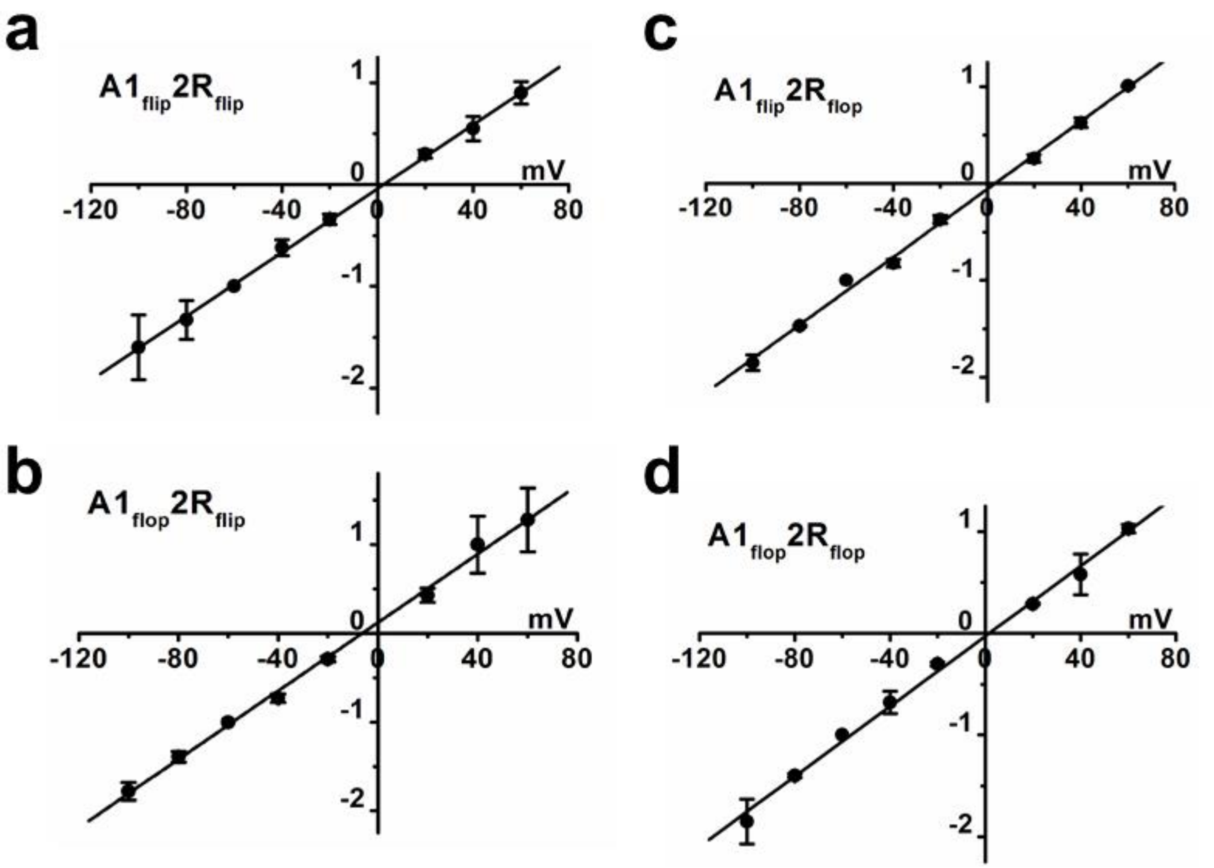

Figure S4. The current-voltage (I-V) relationships of GluA1/2R channels. (a) GluA1$_{flip}$2R$_{flip}$. **(b)** GluA1$_{flop}$2R$_{flip}$. **(c)** GluA1$_{flip}$2R$_{flop}$. **(d)** GluA1$_{flop}$2R$_{flop}$. All whole-cell responses were obtained using 1 mM glutamate as the activating agent. Each data point (±SD) represented the average normalized current amplitude (normalized to the amplitude obtained at -60 mV) from three different cells. GluA2R subunits were edited at the R/G site.

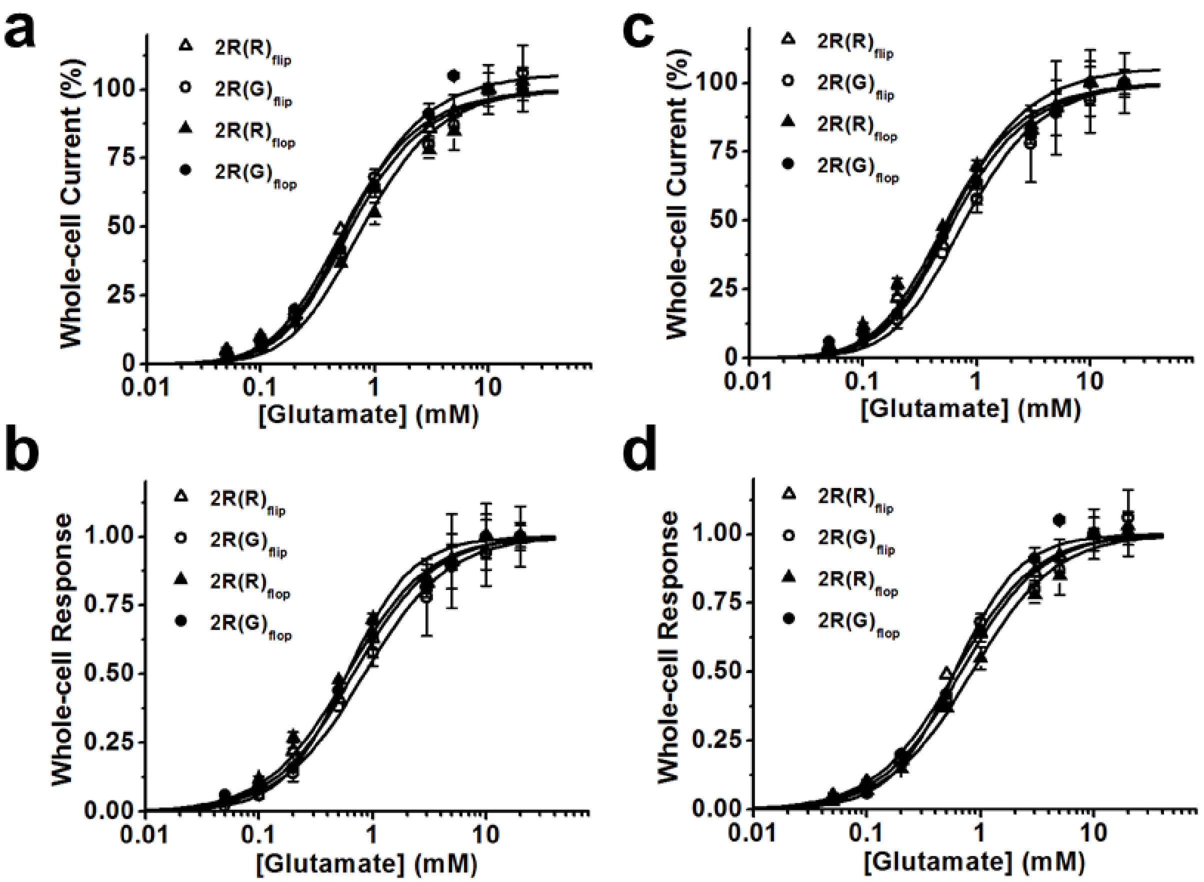

Figure S5. Dose-response fittings of GluA1/2R channels by equation (3) and the Hill Equation, respectively. (a) $GluA1_{flip}2R$ channels, equation (3). **(b)** $GluA1_{flip}2R$ channels, Hill Equation. **(c)** $GluA1_{flop}2R$ channels, equation (3). **(d)** $GluA1_{flop}2R$ channels, Hill Equation. The EC_{50} values are summarized in Tables 3 and 4.

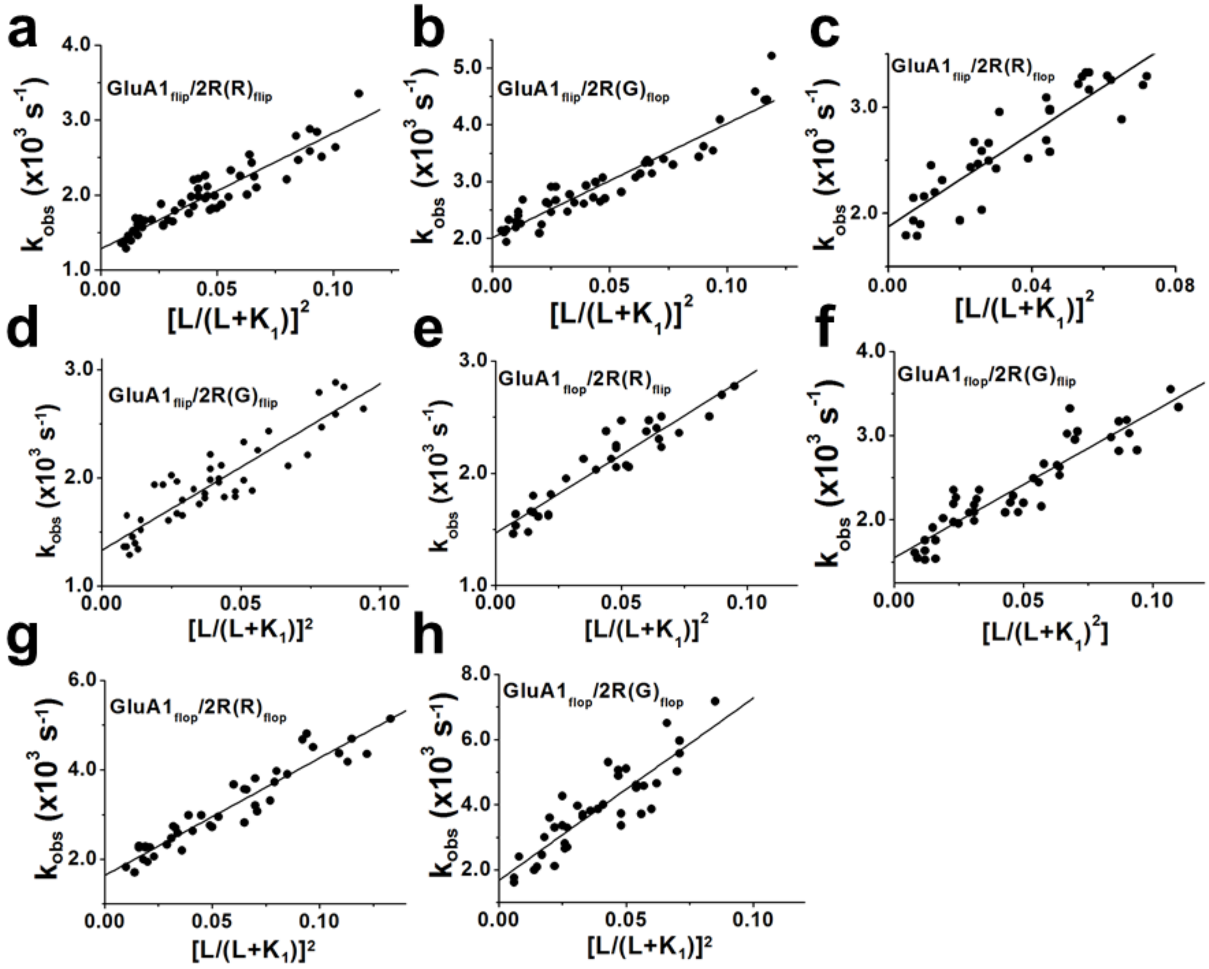

Figure S6. Linear fitting of k_{obs} as a function of glutamate concentration for GluA1/2R channels at n=2.
